# Preschool care early in life and mental health in adolescence in Sweden: a cohort study

**DOI:** 10.1136/bmjopen-2025-105111

**Published:** 2026-04-17

**Authors:** Krisztina D László, Hua Chen, Filip Andersson, Maria Rosaria Galanti

**Affiliations:** 1 Department of Global Public Health, Karolinska Institutet, Stockholm, Sweden; 2 Department of Public Health and Caring Science, Uppsala University, Uppsala, Sweden

**Keywords:** Adolescent, Child, MENTAL HEALTH, Schools

## Abstract

**Abstract:**

**Objective:**

Despite the high rates of early enrolment in preschool and of poor mental health in adolescence in Sweden, knowledge regarding their association in Sweden is lacking. We investigated whether age at starting preschool and weekly hours spent at preschool in different ages are associated with mental ill-health in Swedish adolescents.

**Design:**

A cohort study based on data from KUPOL (Swedish acronym for ‘Knowledge about Adolescents Mental Health and Learning’).

**Setting and participants:**

We used data from KUPOL, a longitudinal study conducted during 2013–2018, involving Swedish adolescents born 2000–2001. We included in the current analyses adolescents with available information on the exposures and the outcomes of interest (n=2261).

**Outcome measures:**

Study participants and their parents completed questionnaires concerning the child’s age (in months) at start of preschool, the average weekly hours in preschool in different ages, the adolescent’s mental health, lifestyle, school-related, psychosocial and parental sociodemographic factors. We analysed the association between preschool-related factors and mental health using logistic regression.

**Results:**

Children enrolled in preschool at the age of 12–15 months had increased odds of high overall and externalising problems score on the Strengths and Difficulties Questionnaire relative to those enrolled at 20 months or later. The corresponding ORs (95% CIs) were 1.39 (95% CI 1.02 to 1.90) and 1.52 (95% CI 1.08 to 2.16), while the corresponding population attributable fractions were 8% and 9%, respectively. There were no associations between age at start of preschool and internalising problems, nor between the average weekly hours at preschool and mental health.

**Conclusion:**

We found weak and inconsistent evidence for a link between early preschool attendance and mental health in adolescence; population attributable fractions suggest limited public health implications for the studied associations. The results should be interpreted in light of the methodological constraints of observational studies, the multitude of our comparisons and the sample selection.

**STRENGTHS AND LIMITATIONS OF THIS STUDY**

- We investigated the association between the age at start of preschool and the time spent at preschool in different ages with several measures of mental health.
- Our study included a contemporary cohort of Swedish children, allowing inference to present circumstances.
- We collected the information on exposure retrospectively, which may lead to some misclassification.
- Although we considered several confounders, we cannot exclude the possibility of residual confounding by unmeasured factors.

## Introduction

 The prevalence of mental health problems in adolescents has increased in many developed countries during the last decades. This increase has been steeper in Sweden than in several other Western societies^1^ and has been reported mainly with respect to mood, anxiety and psychosomatic disorders, some externalising problems and suicide attempts.^2 3^

The reasons for these trends are not clear. Some of the often discussed potential explanations relate to negative changes in the family structure, in the school system, in young adults’ possibilities to enter the labour market and the increased individualisation in the society.^3^ A further substantial societal change in the last decades is the ‘childcare transition’,^4^ that is, the increase in the proportion of women on the labour market during the late 1970s and early 1980s in many high-income countries has led to an increase in the number of children in community care at earlier and earlier ages.^4 5^ In Sweden, the number of children enrolled in daycare has increased consistently from 60 000 in 1975 to almost 500 000 in 2015.^6^

Though the ‘childcare transition’ may be related to several benefits for children, parents and society, concerns have also been expressed regarding the possible association between daycare and socioemotional development.^4 7^ Compelling evidence suggests that the first few years of life represent critical periods of brain development and that the lack of stable, warm and responsive relationships with caregivers early in life may disrupt the development of the brain and of the stress-reactivity^4^ and may result in stress-related illness.^4 8 9^ Several reviews suggest that in the general population, ‘childcare that is ‘too early and for too long’ could be associated with less favourable socioemotional outcomes’.^4 8 9^ Some studies found such associations may persist even until adolescence.^10 11^ Several experts highlight that for many children, high-quality daycare may start becoming a benefit sometime during the third year of life,^4 12^ which coincides with the age when most children develop a better emotional and stress regulation capacity and a stronger interest and ability to interact with peers.^5 12^ High-quality daycare at ages 3–5 years is positively associated with cognitive and social development.^7^

The policies regarding family and daycare are substantially different in Scandinavia than in the countries where most of the studies in this area have been conducted.^13 14^ The 1-year or slightly longer parental leave and the universal daycare access from the age of 1 year have led to group care before this age being almost inexistent in Scandinavian countries. The quality of daycare—measured in terms of the child-to-caregiver ratio and the staff’s educational requirements—is one of the highest in an international perspective.^15^ The generously subsidised daycare fees and several nationwide regulations make the quality of daycares within the country more homogeneous than in many other parts of the world; this may also result in the predictors of daycare utilisation patterns and hence the confounding in studies investigating its health effects, being different in Scandinavia than in countries with other sociopolitical contexts.^16 17^ Regulations that allow parents with small children to reduce their working time and thus possibilities for part-time daycare attendance arrangements tailored to each family’s needs are also outstanding in an international perspective. The high proportion of women and men in the labour market and the existence of a high quality, subsidised and universally accessible daycare system contributed to the rate of children under the age of 3 years enrolled in daycare being among the highest in the Nordic countries.^18^ In Sweden, around 83% of children aged 1–5 years attended preschool^19^; half of those aged 1 year are enrolled, while rates are ≈90% at age 2 years and ≈95% at age 3 years.^5 18^ In light of the substantial contextual differences, the findings and the public health implications of the studies investigating the association between different characteristics of daycare and mental health, conducted predominantly in North America and the UK, may not be generalisable to Scandinavian countries.

Despite the high rate of children under 2 years in daycare,^4^ few Nordic studies have analysed the associations between attending preschool at early ages and the time at preschool with children’s socioemotional development and their findings have been mixed. A Danish and a few Norwegian studies found modest positive or no associations of early age at daycare enrolment (defined often as <18 months) and/or weekly time in daycare with distress and externalising behavioural problems assessed in preschool or primary school.^1317 20^,^25^ To our knowledge, only a few studies investigated this question in a Swedish context and their findings have been mixed,^1626^,^29^ possibly due to differences in the characteristics of the study population, as well as in the definition of exposure and outcome. All three studies were small (n=52 to 140) and none has followed children further than early teenage, when the rise in rates of internalising problems becomes apparent. In addition, given the negative changes in group size and the child-to-staff ratio following the economic crisis of the early 1990s, as well as in the out-of-home care curriculum of the children aged 1–5 years in 1998, generalisability of these early Swedish studies to more recent daycare-related circumstances is unclear.^5^

The aim of this study was to analyse whether the age at start of preschool and the average weekly hours at preschool are associated with mental ill-health among adolescents in Sweden. We hypothesised that an early start and a high number of weekly hours spent at preschool may increase the risk of poor mental health in adolescence.

## Methods

### Data source and study population

#### Data source

The study was based on KUPOL (Swedish acronym for ‘Knowledge about Adolescents Mental Health and Learning’), a longitudinal cohort study involving adolescents born 2000–2001 from eight regions in Sweden.^30^ Briefly, 541 secondary schools from central and southern Sweden, each having at least 20 students in each grade of the upper part of the primary school (ie, grades 7–9) were asked to take part in the KUPOL study. The schools that accepted to participate (n=101, response rate at school level: 19%) provided students in the seventh grade in the academic years 2013/2014 and 2014/2015 (n=12 512) and their families written information about the KUPOL study. Legal guardians of 3959 children (response rate at child level: 32%) gave written informed consent for participation in KUPOL. Data for wave 1 were collected when children were in grade 7, for wave 2 in grade 8, for wave 3 in grade 9 and for wave 4 in grade 10. Adolescents and their parents completed on each occasion extensive questionnaires concerning sociodemographic, school-related, lifestyle and psychosocial factors. We obtained further information from population-based registers for 3517 children, that is, for whom we had written consent for register data retrieval.

#### Study population

Analyses for the current study were performed among adolescents whose parent responded to at least one question related to the child’s preschool attendance and who had information on mental health from at least one wave in KUPOL (n=2261, ie, 64% of the 3517). A flowchart of the participation in the study is included in the online supplemental figure S1.

### Study variables

#### Exposure

Information on preschool attendance was collected through the questionnaire sent to the legal guardians in wave 4. We studied two main exposures, that is,(1) the age when the child started preschool or daycare (in months) and (2) the average weekly hours the child was at preschool or daycare between the ages 1–2 years, 2–3 years and 3–6 years, with the answer possibilities for each age range being: up to 15 hours, 15–34 hours, ≥35 hours and ‘I do not remember’. For the main analyses, we categorised the age when the child started preschool or daycare (in months) based on the tertile distribution as 12–15 months, 16–19 months and ≥20 months.

#### Outcomes

Adolescents’ mental health was measured on four occasions using the self-reported version of the Strengths and Difficulties Questionnaire (SDQ) and the Center for Epidemiological Studies Depression Scale for Children (CES-DC). The SDQ consists of 25 items that are grouped in five scales that assess emotional symptoms, hyperactivity/inattention symptoms, conduct problems, peer problems and prosocial behaviour; responses are given on a three-point scale, that is, not true (0), somewhat true (1) and certainly true (2).^31^ We calculated the total problem score by summing the responses on the 25 items, the internalising problem score by summing scores on the emotional symptoms and the peer problems scales, and the externalising problem score by summing answers on the hyperactivity/inattention and the conduct problems scales.^32^ We categorised the scales based on the cut-offs suggested by Goodman *et al*,^32^ that is, <18 versus ≥18 in case of the total difficulties score; <9 versus ≥9 in case of the internalising problems scale and <11 versus ≥11 in case of the externalising problems scale. The CES-DC includes 20 items and is used to screen for symptoms of depression among children and adolescents. Study participants rate on a scale ranging from 0 to 3 the frequency with which they experienced certain depressive symptoms in the past week. We calculated the total score by summing the responses on the individual items and categorised the variable as <30 versus ≥30, as previously suggested.^33^ We categorised each outcome as scoring above the recommended cut-off at least on one of the four occasions versus never.

#### Covariates

Information on parents’ education and country of birth, maternal age, child’s gender, paternal and maternal income and employment status, gestational age, birth weight, singleton/multiple birth, child’s congenital malformations, the quality of the parent-child relationship, confidence in others, peer network, family support and academic achievement was retrieved from questionnaires or registers, as described in the online supplemental material.

### Statistical analysis

We compared exposure groups by means of χ^2^ tests in case of categorical variables and by means of Kruskal-Wallis tests in case of continuous variables with non-normal distribution. We investigated the associations between the main preschool-related variables (ie, age at start of preschool and time spent at preschool/week) and each mental health outcome (ie, score above the cut-off on the total SDQ scale, SDQ internalising problems, SDQ externalising problems and CES-DC, respectively) using logistic regression. We adjusted for maternal age at child’s birth, parental country of origin, education, income and employment in the child’s first year of life, the child’s gestational age, birth weight, being singleton and having a congenital malformation. The choice of confounders was based on (1) their known or theoretically plausible association with both the preschool-related variables and mental health and (2) not being on the causal pathway between exposure and outcome. We assessed the model’s assumptions for all main logistic regression analyses. We evaluated multicollinearity among covariates using generalised variance inflation factors; we did not find evidence of high multicollinearity. We also conducted influence diagnostic tests; we did not find influential observations. We estimated ORs and corresponding 95% CIs and adjusted predictive probabilities. If we observed associations in the primary analyses, we also calculated the corresponding population attributable fraction. In the case of missing covariates, in the main analyses, we applied listwise deletion.

When we observed an association, we further adjusted for factors that may be on the pathway between exposure and outcome, that is, the warmth of the parent-child relationship, confidence in others, number of friends, family support and academic grades (one-by-one, all measured in wave 1) to investigate whether these factors may contribute to the explanation of the association of interest. To investigate whether the association of age at enrolment and time spent at preschool with mental health varies by the gender of the child (boy vs girl), maternal age when the child was born (categorised ≤30 years versus ≥31 years) and maternal education (0–14 years versus ≥15 years), we performed stratified analysis and formal tests of interaction with these variables.

We performed several sensitivity analyses. First, to balance baseline characteristics between exposure groups, we ran analyses with propensity score matching (PSM). Second, to analyse whether our categorisation of age at start of preschool influenced the results, we repeated our main models with the variable treated as (1) continuous and (2) dichotomised at the median, that is, at 18 months. Third, we repeated our main analyses with the outcome treated as a continuous variable; we used linear regression to generate and compare least square means. Fourth, we imputed missing covariates using multiple imputation (20 imputations) and pooled the results using Rubin’s rules.

All tests were two-sided, with α=0.05.

SAS V.9.4 (SAS Institute) and R V.4.4 were used for the analyses.

## Results

Of 2261 children whose parent provided information about the age when the child started preschool, 642 started at the age of 12–15 months, 869 at the age of 16–19 months and 750 at ≥20 months. Characteristics of the cohort by age at preschool enrolment are shown in table 1.

**Table 1:** Characteristics of study participants according to age of enrolling in preschool

Characteristics	Total (n=2261)	Age of the child when starting preschool (months)			
		12–15 (n=642)	16–19 (n=869)	≥20 (n=750)	P value*
Categorical variables, n (%)					
Child’s gender					0.99
Boy	1065 (47.1)	302 (47.0)	409 (47.1)	354 (47.2)	
Girl	1196 (52.9)	340 (53.0)	460 (52.9)	396 (52.8)	
Mother’s age when the child was born (years)					<0.01
≤25	195 (8.6)	71 (11.1)	77 (8.9)	47 (6.3)	
26–30	719 (31.8)	196 (30.5)	315 (36.2)	208 (27.7)	
31–35	759 (33.6)	217 (33.8)	280 (32.2)	262 (34.9)	
36–40	315 (13.9)	78 (12.1)	115 (13.2)	122 (16.3)	
≥41	62 (2.7)	12 (1.9)	19 (2.2)	31 (4.1)	
Missing	211 (9.3)	68 (10.6)	63 (7.2)	80 (10.7)	
Both parents were born in Sweden					0.02
Yes	1736 (76.8)	466 (72.6)	688 (79.2)	582 (77.6)	
No	317 (14.0)	109 (17.0)	106 (12.2)	102 (13.6)	
Missing	208 (9.2)	67 (10.4)	75 (8.6)	66 (8.8)	
Paternal education level (years)					0.45
0–9	150 (6.6)	49 (7.6)	53 (6.1)	48 (6.4)	
10–14	1479 (65.4)	425 (66.2)	559 (64.3)	495 (66.0)	
≥15	621 (27.5)	166 (25.9)	256 (29.5)	199 (26.5)	
Missing	11 (0.5)	2 (0.3)	1 (0.1)	8 (1.1)	
Maternal educational level (years)					0.32
0–9	79 (3.5)	30 (4.7)	30 (3.5)	19 (2.5)	
10–14	1347 (59.6)	380 (59.2)	516 (59.4)	451 (60.1)	
≥15	828 (36.6)	231 (36.0)	322 (37.1)	275 (36.7)	
Missing	7 (0.3)	1 (0.2)	1 (0.1)	5 (0.7)	
Paternal employment status in the child’s first year of life					<0.01
Employed	2033 (89.9)	578 (90.0)	813 (93.6)	642 (85.6)	
Unemployed	126 (5.6)	41 (6.4)	28 (3.2)	57 (7.6)	
Missing	102 (4.5)	23 (3.6)	28 (3.2)	51 (6.8)	
Maternal employment status in the child’s first year of life					0.49
Employed	1732 (76.6)	493 (76.8)	684 (78.7)	555 (74.0)	
Unemployed	435 (19.2)	126 (19.6)	159 (18.3)	150 (20.0)	
Missing	94 (4.2)	23 (3.6)	26 (3.0)	45 (6.0)	
Paternal income in the child’s first year of life					0.02
Low tertile	710 (31.4)	213 (33.2)	243 (28.0)	254 (33.9)	
Middle tertile	728 (32.2)	214 (33.3)	297 (34.2)	217 (28.9)	
High tertile	721 (31.9)	192 (29.9)	301 (34.6)	228 (30.4)	
Missing	102 (4.5)	23 (3.6)	28 (3.2)	51 (6.8)	
Maternal income in the child’s first year of life					<0.01
Low tertile	712 (31.5)	221 (34.4)	234 (26.9)	257 (34.3)	
Middle tertile	737 (32.6)	211 (32.9)	308 (35.4)	218 (29.1)	
High tertile	718 (31.8)	187 (29.1)	301 (34.6)	230 (30.7)	
Missing	94 (4.2)	23 (3.6)	26 (3.0)	45 (6.0)	
Gestational age (weeks)					<0.01
<32	12 (0.5)	0	5 (0.6)	7 (0.9)	
32–36	106 (4.7)	22 (3.4)	34 (3.9)	50 (6.7)	
≥37	1953 (86.4)	575 (89.6)	771 (88.7)	607 (80.9)	
Missing	190 (8.4)	45 (7.0)	59 (6.8)	86 (11.5)	
Birth weight (g)					0.01
<2500	74 (3.3)	13 (2.0)	26 (3.0)	35 (4.7)	
≥2500	1994 (88.2)	581 (90.5)	784 (90.2)	629 (83.9)	
Missing	193 (8.5)	48 (7.5)	59 (6.8)	86 (11.5)	
Singleton					<0.01
No	75 (3.3)	12 (1.9)	24 (2.8)	39 (5.2)	
Yes	2186 (96.7)	630 (98.1)	845 (97.2)	711 (94.8)	
Congenital malformation					0.02
Yes	65 (2.9)	28 (4.4)	18 (2.1)	19 (2.5)	
No	2196 (97.1)	614 (95.6)	851 (97.9)	731 (97.5)	
Number of male friends					0.35
0	302 (13.4)	80 (12.5)	110 (12.7)	112 (14.9)	
1	249 (11.0)	61 (9.5)	109 (12.5)	79 (10.5)	
2	314 (13.9)	93 (14.5)	122 (14.0)	99 (13.2)	
≥3	1352 (59.8)	399 (62.1)	513 (59.0)	440 (58.7)	
Missing	44 (1.9)	9 (1.4)	15 (1.7)	20 (2.7)	
Number of female friends					0.83
0	215 (9.5)	62 (9.7)	87 (10.0)	66 (8.8)	
1	197 (8.7)	52 (8.1)	77 (8.9)	68 (9.1)	
2	317 (14.0)	82 (12.8)	129 (14.8)	106 (14.1)	
≥3	1481 (65.5)	433 (67.4)	557 (64.1)	491 (65.5)	
Missing	51 (2.3)	13 (2.0)	19 (2.2)	19 (2.5)	
Sum of grades in Swedish, English and Mathematics (grade 7)					0.02
<35	292 (12.9)	91 (14.2)	101 (11.6)	100 (13.3)	
35–50	1424 (63.0)	401 (62.5)	538 (61.9)	485 (64.7)	
>50	384 (17.0)	92 (14.3)	176 (20.3)	116 (15.5)	
Missing	161 (7.1)	58 (9.0)	54 (6.2)	49 (6.5)	
Continuous variables, median					
Maternal warmth as rated by the child	16	16	16	16	0.02
Paternal warmth as rated by the child	16	15	16	16	0.03
Parental warmth as rated by the parent	17	17	17	17	0.79
Confidence in others	7	7	8	8	0.01
Family support in school-related issues	20	19	20	20	0.06

* P values were estimated using χ2 tests for categorical variables and Kruskal-Wallis tests for continuous variables.

The numbers and rates of high total SDQ score, internalising problems and externalising problems in at least one of the four waves were 447 (19.8%), 666 (29.5%) and 332 (14.7%), respectively. Children who started preschool at 12–15 months had an increased odds of high total SDQ and externalising problems scores relative to those who started preschool at the age of ≥20 months; the corresponding adjusted ORs (95% CIs) were 1.39 (95% CI 1.02 to 1.90) and 1.52 (95% CI 1.08 to 2.16), respectively (table 2). Absolute risk differences for these associations were 5% and 6%, while corresponding population attributable fractions were 8% and 9%, respectively. There were no substantial differences in these outcomes between those who started daycare at 16–19 months and ≥20 months, nor was there a link between age at preschool enrolment and scores on the internalising problems scale. There was no association between the average weekly hours the child was in preschool in different ages and the total SDQ, SDQ internalising or externalising problems; children whose parents did not remember the time the child was at preschool had increased odds of these outcomes. The adjusted predicted probabilities corresponding to these analyses are shown in online supplemental table S1. The number and rate of adolescents with high CES-DC in at least one of the waves was 583 (25.8%). Age at starting and time at preschool in different ages was not associated with depressive symptoms; children whose parents did not remember the time amount the child spent at preschool had an increased odds of depressive symptoms (table 3); corresponding adjusted predicted probabilities are also shown in online supplemental table S1.

**Table 2:** ORs and 95% CIs for the association between preschool-related variables and mental health as measured by the SDQ

Type of exposure	Total n (%)	SDQ total score		SDQ internalising problems		SDQ externalising problems	
		Events	Adjusted OR (95% CI)*	Events	Adjusted OR (95% CI)*	Events	Adjusted OR (95% CI)*
The age when the child started preschool (n=2261)							
12–15 months	642 (28.4)	150	1.39 (1.02 to 1.90)	196	1.09 (0.83 to 1.43)	120	1.52 (1.08 to 2.16)
16–19 months	869 (38.4)	163	1.10 (0.82 to 1.49)	255	1.02 (0.79 to 1.32)	112	0.99 (0.70 to 1.39)
≥20 months	750 (33.2)	134	1.00	215	1.00	100	1.00
Time spent at preschool in different ages (hours/week)							
Time spent at preschool between the ages of 1 year and 2 years (n=1779)†							
Up to 15 hours/week	271 (15.2)	51	1.00	84	1.00	46	1.00
15–34 hours/week	1090 (61.3)	208	1.09 (0.73 to 1.63)	312	0.85 (0.61 to 1.20)	147	0.84 (0.55 to 1.28)
≥35 hours/week	323 (18.2)	67	1.09 (0.68 to 1.77)	94	0.87 (0.58 to 1.31)	45	0.65 (0.38 to 1.13)
Does not remember	95 (5.3)	27	2.14 (1.11 to 4.10)	35	1.28 (0.70 to 2.34)	26	2.16 (1.11 to 4.20)
Time spent at preschool between the ages of 2 years and 3 years (n=2056)‡							
Up to 15 hours/week	275 (13.4)	49	1.00	71	1.00	44	1.00
15–34 hours/week	1231 (59.9)	227	1.11 (0.75 to 1.64)	359	1.05 (0.75 to 1.47)	160	0.93 (0.61 to 1.43)
≥35 hours/week	471 (22.9)	107	1.39 (0.90 to 2.15)	146	1.20 (0.83 to 1.76)	77	1.04 (0.64 to 1.70)
Does not remember	79 (3.8)	29	3.52 (1.83 to 6.78)	31	1.84 (0.98 to 3.46)	25	3.38 (1.71 to 6.67)
Time spent at preschool between the ages of 3 years and 6 years (n=2208)§							
Up to 15 hours/week	119 (5.4)	22	1.00	40	1.00	14	1.00
15–34 hours/week	1180 (53.4)	208	0.88 (0.51 to 1.51)	333	0.86 (0.54 to 1.38)	155	1.29 (0.66 to 2.52)
≥35 hours/week	833 (37.7)	182	1.32 (0.76 to 2.29)	247	0.97 (0.60 to 1.57)	129	1.76 (0.89 to 3.47)
Does not remember	76 (3.4)	25	2.46 (1.14 to 5.34)	30	1.45 (0.71 to 2.99)	23	4.09 (1.71 to 9.76)

* Adjusted for maternal age at child’s birth, parental country of origin, education, income and employment in the child’s first year of life and the child’s gestational age, birth weight, being singleton and having a congenital malformation; analyses were restricted to participants with complete data on the exposure, outcome and adjusted covariates.

† Analyses included study participants who started preschool between the ages of 1–2 years.

‡ Analyses included study participants who started preschool before the age of 3 years.

§ Analyses included study participants who had been at preschool.

CI, confidence interval; OR, odds ratio; SDQ, Strengths and Difficulties Questionnaire.

**Table 3:** ORs and 95% CIs for the association between preschool-related variables and depression as measured by the CES-DC

Type of exposure*	Total n (%)	Depression measured by CES-DC	
		Events	Adjusted OR (95% CI)†
The age when the child started preschool (n=2261)			
12–15 months	642 (28.4)	175	1.00 (0.76 to 1.33)
16–19 months	869 (38.4)	210	0.91 (0.70 to 1.19)
≥20 months	750 (33.2)	198	1.00
Time spent at preschool in different ages (hours/week)			
Time spent at preschool between the ages of 1 year and 2 years (n=1779)*			
Up to 15 hours/week	271 (15.2)	71	1.00
15–34 hours/week	1090 (61.3)	269	0.96 (0.67 to 1.37)
≥35 hours/week	323 (18.2)	76	0.87 (0.56 to 1.34)
Does not remember	95 (5.3)	33	1.52 (0.82 to 2.80)
Time spent at preschool between the ages of 2 years and 3 years (n=2056)‡			
Up to 15 hours/week	275 (13.4)	63	1.00
15–34 hours/week	1231 (59.9)	304	1.06 (0.74 to 1.50)
≥35 hours/week	471 (22.9)	126	1.17 (0.78 to 1.74)
Does not remember	79 (3.8)	27	2.25 (1.20 to 4.24)
Time spent at preschool between the ages of 3 years and 6 years (n=2208)§			
Up to 15 hours/week	119 (5.4)	31	1.00
15–34 hours/week	1180 (53.4)	288	0.84 (0.51 to 1.37)
≥35 hours/week	833 (37.7)	218	1.05 (0.64 to 1.74)
Does not remember	76 (3.4)	29	2.23 (1.08 to 4.60)

* Analyses included study participants who started preschool between the ages of 1–2 years.

† Adjusted for maternal age at child’s birth, parental country of origin, education, income and employment in the child’s first year of life and the child’s gestational age, birth weight, being singleton and having a congenital malformation; analyses were restricted to participants with complete data on the exposure, outcome and adjusted covariates.

‡ Analyses included study participants who started preschool before the age of 3 years.

§ Analyses included study participants who had been at preschool.

CES-DC, Centre for Epidemiological Studies Depression Scale for Children; CI, confidence interval; OR, odds ratio.

Adding parental warmth, confidence in others, numbers of friends, family support in school-related issues and academic results to the confounder-adjusted models did not change the association between age at preschool enrolment and the total SDQ (online supplemental table S2). We found no major, consistent differences in the association between age at preschool enrolment and time spent in daycare and high SDQ total score and CES-DC according to the child’s gender, maternal age or maternal education (data not shown). There was some evidence for a stronger association between spending an increased number of hours at preschool and increased odds of high total SDQ score and CES-DC among children of parents aged ≤30 years than among those of parents ≥31 years (data not shown).

The results of the PSM analyses were largely consistent with those from the main multivariable logistic regression; the observed differences between groups were null or weak (online supplemental table S3). We observed no association between age at start of preschool treated as a continuous variable and the four mental health outcomes (online supplemental table S4). When age at start of preschool was dichotomised, results were generally similar to those observed in the primary analyses (online supplemental table S5). The adjusted mean differences in total SDQ scores, SDQ internalising and externalising problem scores and total CES-DC scores across exposure groups were generally small (online supplemental table S6). Results were largely consistent with those in the main analyses when using multiple imputation for missing covariates (online supplemental table S7).

## Discussion

Swedish children who started preschool at the age of 12–15 months had higher odds of high total SDQ and externalising problems scores than those who started preschool at 20 months or later; the strength of the associations was very modest and the corresponding absolute risk differences and the population attributable fractions were low. There was no association between the age at start of preschool and the odds of internalising problems, nor between the number of hours spent at preschool in different ages and the odds of mental ill-health.

A large, though not consistent, body of evidence—predominantly from studies from the USA, Australia, Canada and UK—suggests that too early and too extensive out of home care may be negatively related to children’s socioemotional development and mental health.^4 8 9^ Given important differences between countries in policies regarding family and childcare, and thus also in the determinants of use of daycare and in the confounders of its association with mental health, cross-cultural generalisability of these earlier findings may be limited. Despite the high rates of enrolment in childcare before the age of 2 years and the fact that children younger than two spend on average 32 hours per week in community group care,^18^ only a few Swedish studies investigated the association between age at start and time at preschool and the child’s socioemotional development and their findings have been mixed. Andersson reported a positive association between the start of daycare between the ages of 6–12 months and socioemotional competence in the school at the ages of 8 years^26^ and 13 years.^27^ A study from Gothenburg found that the number of months in daycare before the age of 2 years and the number of hours in daycare were negatively associated with compliance with maternal demands at 40 months^28^; there was no association between daycare-related factors and compliance and aggression at 80 months.^29^ A study by Hagekull and Bohlin found no association between age at entry in daycare and internalising or externalising problems at the age of 4 years.^16^ The explanations for the inconsistent findings of these earlier Swedish studies are not clear, but besides differences in applied measures, age at assessing the outcome and considered confounders, socioeconomic characteristics of the sample may account for the differences in results. Children from Anderson’s study were recruited from low-resource and middle-resource neighbourhoods and the study heavily oversampled single mothers (1/3).^26 27^ The recruitment base in the Gothenburg project was the municipal waiting list for a daycare place, which in the 1980s, when the study was conducted, is likely to have contained a better-off population.^28^ Selection criteria in Hagekull and Bohlin’s study might have also favoured a slightly better-off category. Our findings extend those of these earlier studies by including a contemporary cohort of Swedish children, a substantially larger sample and probably somewhat more diverse than that of the previous studies, follow-up up to the age of 16 years and investigating the association between the age at start of preschool and the time spent at preschool in different ages with several dimensions of mental health. Overall, we observed limited and somewhat inconsistent evidence for an association between preschool attendance early in life and mental health.

We found associations only between enrolment to preschool at 12–15 months and the odds of high SDQ total and externalising problems score, but not in the case of internalising problems. The associations seen for externalising problems are likely to drive also that with the total SDQ score. There are several potential explanations for these associations.^10^,^1234^ A first major explanation—as hypothesised—is that the early separation from parents may negatively influence the parent-child attachment, the architecture of the developing brain^35^ and the programming of stress reactivity.^36 37^ These factors could impact compliance with parental demands,^38^ the child’s capacity to regulate emotions and behaviour,^39 40^ the ability to form and maintain long-term intimate relationships,^41^ cognitive development^42^ and substance use later in life.^43^ These, in turn, may increase the risk of poor mental health. We speculate that our observation that an association between an early start of daycare and poor mental health was observed primarily in the case of externalising problems may suggest that early separation from parents may be accompanied by an excessive attention-seeking behaviour and emotional and behavioural regulation difficulties, which in turn may increase the risk of externalising problems. We investigated several possible mechanisms that may link our preschool-related variables to mental health, but we found that none of these characteristics contributed to the explanation of the association between preschool enrolment age and mental health. Possible explanations for these findings may be that some of the potential mediators were assessed close in time to the outcome or that other, unmeasured characteristics may be of importance.

Several methodological aspects may further contribute to the explanations of these positive but weak associations. The first one is selection. Parents who choose to enrol their children in daycare at early ages and their children may differ from their counterparts enrolling in daycare in later ages on personal characteristics and contextual factors that make it difficult to make predictions on the magnitude and directions of the potential bias. For example, some parents may have chosen an early enrolment in daycare because of the child’s behavioural problems (eg, frequent crying or being hyperactive) and/or because of the parent himself/herself having poor mental health. On the other hand, socially well-integrated parents with progressive parenting values may send their children to daycare earlier than parents who do not endorse trust in the community services; the former may also be more tolerant to externalising behaviours in the child. Despite the rather comprehensive attempt to control for confounders, we cannot exclude the possibility that the weak association between start of daycare at the age of 12–15 months and the odds of high SDQ and high externalising problems in adolescence may be explained by confounding. Second, if the association between early start of daycare and the risk of high SDQ and high externalising problems was causal, we could expect a dose-response association between (1) an early age at starting daycare and (2) an increasing number of hours spent at daycare, especially between the ages 1–2 years and the risks of these outcomes. The lack of a dose-response pattern does not provide support for a causal interpretation. However, some parents may have a short-term flexibility to adopt the amount of time the child is at preschool according to the child’s socioemotional reactions and self-regulatory capacity, which in turn may buffer negative effects on mental health. Descriptive figures in tables2  3 suggest that the time amount children spent at preschool increased with age, that is, that child’s age and associated socioemotional maturity affect parents’ choice regarding the time amount they use preschool services. Third, given the multitude of tests, some of the observed statistically significant associations could be due to chance. Nevertheless, though the number of performed tests was high, it is important to note that analyses were driven by hypotheses formulated before the data collection and that the high number of analyses was ‘clustered’ around the hypotheses, outcomes and exposures; when relevant, the different sets of analyses are used to scrutinise if they confirm each other. For example, the SDQ internalising problems scale and the CES-DC capture similar aspects, while the SDQ total score is calculated based on information on the SDQ internalising and externalising scales. Similarly, the three large sets of analyses according to the time spent at preschool (in ages 1–2 years, 2–3 years and 3 years and 6 years) are also strongly inter-related; if there is no association with high number of hours at preschool in ages 1–2 years, it may be expected that there will be no association later either.

Several limitations of the study need to be considered. First, since parents provided the information on the preschool-related characteristics retrospectively, some misclassification of our exposures is possible. We attempted to reduce the biases associated with the retrospective exposure assessment by using data from different informants on exposures and outcomes. While parents are likely to relatively accurately recall the child’s age when they enrolled him/her in preschool, misclassification may be more important for the number of hours the child spent at preschool in different ages. To address this concern, we offered large interval-based answer possibilities (ie, up to 15 hours/week, 15–34 hours/week and 35 hours/week or more hours/week) and the ‘I do not know’ option. When deciding on the above cut-offs, we considered that several municipalities regulate the maximal amount of hours per week parents on parental leave with a younger sibling or unemployed parents may have their children at preschool (15 hours/week or 30 hours/week). Second, given that the non-response rate at different recruitment stages (school, individual and wave) was high and the participation in the study varied by parental sociodemographic characteristics (ie, the rate of children with parents born in Sweden and with high education was higher than that corresponding to that in the study base),^30^ the non-response may affect external validity. Given the low overall response rate and that the sample was selected, it is not clear to what extent our findings may be generalised to the total population of Swedish children. Several earlier studies have suggested that there may be some benefit for enrolling early in a high-quality daycare for children from disadvantaged as opposed to those from more advantaged families,^7^ suggesting that the association between early enrolment in daycare and mental health varies by parental socioeconomic status. Given the characteristics of the participants in this study, it is plausible that the adverse associations observed are overestimated compared with what we would have observed in the total population where a substantial proportion of children would have been affected positively by early enrolment in daycare. Nevertheless, stratified analyses by parental education and country of birth did not suggest evidence of effect modification by these factors. Fourth, though our sample size was large, in some analyses we may have lacked statistical power to identify modest associations. Fifth, the estimates of the population attributable fraction should be regarded as indicative and not definitive, given its strong requirements regarding causality and unbiased estimates.

In conclusion, we found weak and inconsistent evidence for a link between early preschool attendance and mental health in adolescence. There was some evidence that children enrolled in preschool at 12–15 months had modestly increased odds of high total SDQ and externalising problems scores relative to children who started preschool at 20 months or later; nevertheless, corresponding population attributable fractions suggest limited public health implications for these associations. There was no evidence of a link between age at preschool enrolment and the increased odds of internalising problems nor of an association between the number of hours spent at preschool in different ages and the increased odds of mental ill-health. Our results need to be interpreted in light of the methodological constraints of observational studies, the multitude of comparisons and the sample selection.

## Supplementary material

10.1136/bmjopen-2025-105111 online supplemental file 1

## Data Availability

Data are not publicly available.
