# Supplementary material for: Preschool care early in life and mental health in adolescence in Sweden: a cohort study
Source: BMJ Open. 2026 Apr 17;16(4):e105111. doi: 10.1136/bmjopen-2025-105111 (PMC13110637; doi:10.1136/bmjopen-2025-105111)
Supplement: online supplemental file 1 [file bmjopen-16-4-s001.docx]

**Supporting information**

*Covariates*

Data on parents’ education and country of birth and maternal age was obtained from the questionnaire completed by the parent in wave 1, or if this information was missing, from later waves. We dichotomized parental education as 0-9, 10-14 and ≥15 years of education. Parent’s country of birth was dichotomized as both parents born in Sweden versus at least one of the parents was born outside Sweden. Maternal age was categorized as ≤25, 26-30, 31-35, 36-40 and ≥41 years. Information on the child’s gender was extracted from their personal number. Information on paternal and maternal income and employment status during the year after childbirth was obtained from The Longitudinal Database for Health Insurance and Labor Market Studies; the income variables were categorized based on their tertile distribution. Information on gestational age, birth weight, singleton/multiple birth and the child’s congenital malformations was retrieved from the Medical Birth Register and categorized as shown in Table 1.

The quality of the parent-child relationship was assessed using the parent- and the child-reported versions of the Parental Warmth Scale.^1^ The questionnaire completed by the child asks how often the mother and the father, respectively exhibit gestures indicative of care, pride and love.^1^ In the questionnaire’s parent-version the parent rates how often he/she exhibits such gestures. The scale consists of six items rated from 1 to 3, with the total score being calculated as the sum of the individual item scores. Confidence in others was assessed by two items (“In general, most people can be trusted” and “Most people are fair and don’t take advantage of you”) scored from strongly disagree (1) to strongly agree (5). Peer network was assessed using the items “At present, how many close male/female friends do you have?”. Family support in school-related issues was assessed by three items (“My family is there for me when I need them”, “When I have problems at school my family is willing to help” and “When something good happens at school, my family wants to know about it”) rates from “strongly disagree” (1) to “strongly agree” (5). We used as an indicator of academic achievement the sum of the student-reported academic grades in Swedish, English and Mathematics in the seventh grade. Each subject is rated from 0 to 20; we categorized the total score as <35, 35-50 and >50. Information on the potential mediators was from wave 1; in case of data from wave 1 was missing, we used information from the subsequent available wave.


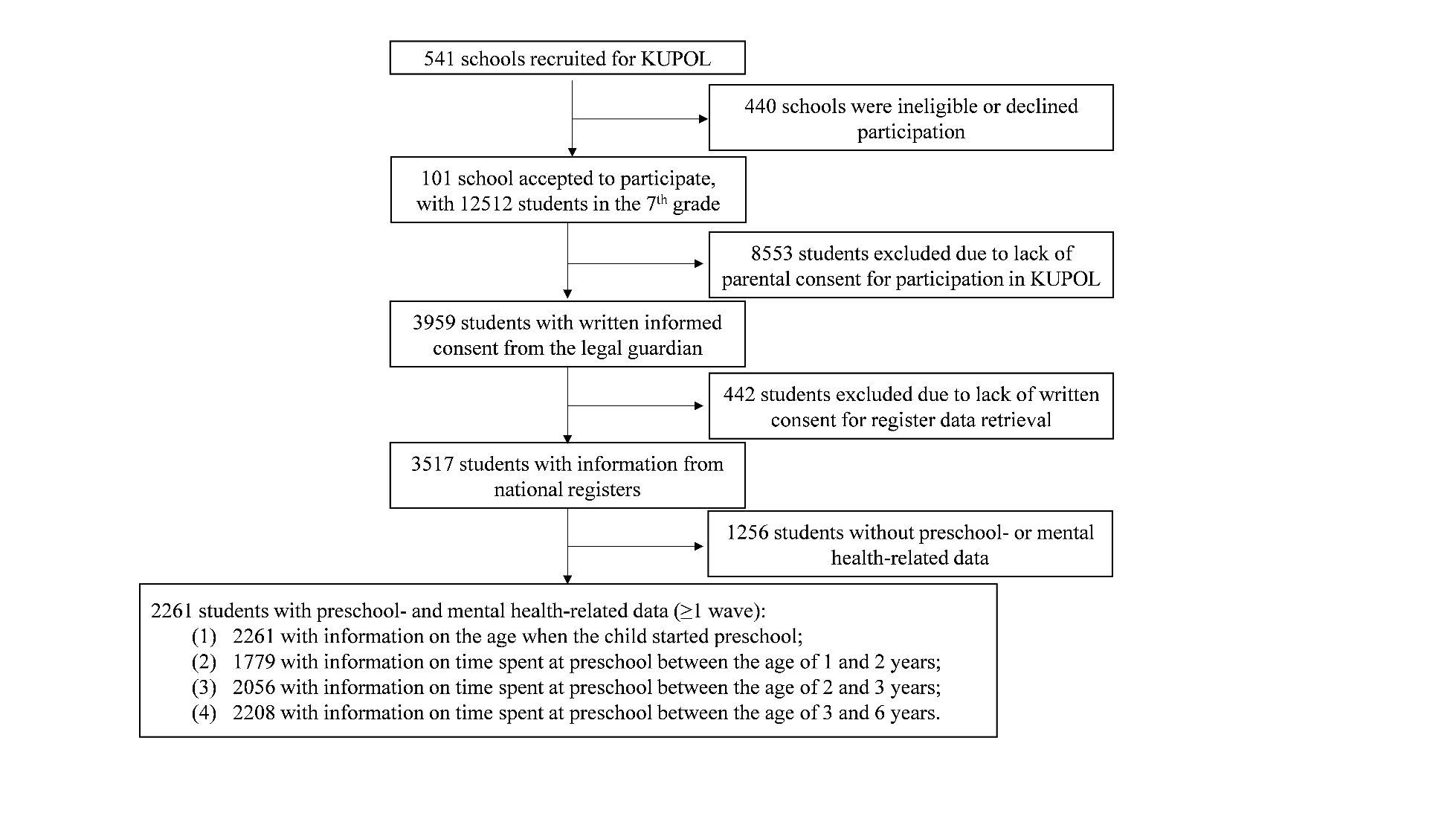


Figure S1 Flow chart for participation in the study. KUPOL = Swedish acronym for “Knowledge about Adolescents Mental Health and Learning”.

**Table S1** Adjusted predicted probabilities and 95% confidence intervals of poor mental health outcomes by preschool-related variables

| **Type of exposure** | **Adjusted predicted probability, % (95% CI)^a^** | | | |
| --- | --- | --- | --- | --- |
|  | **SDQ total score** | **SDQ internalizing problems** | **SDQ externalizing problems** | **Depression measured by CES-DC** |
| ***The age when the child started preschool (n=2261)*** | |  |  |  |
| 12-15 months | 19 (8-37) | 24 (13-39) | 16 (8-28) | 22 (12-38) |
| 16-19 months | 15 (7-32) | 23 (13-37) | 11 (5-20) | 21 (11-36) |
| ≥20 months | 14 (6-29) | 22 (12-37) | 11 (5-21) | 22 (12-38) |
| ***Time spent at preschool in different ages (hours/week)*** | | |  |  |
| *Time spent at preschool between the ages of 1 and 2 years (n=1779)^b^* | | |  |  |
| Up to 15 hours/week | 12 (4-29) | 25 (12-45) | 11 (4-24) | 23 (11-42) |
| 15-34 hours/week | 13 (5-30) | 22 (11-40) | 9 (4-20) | 22 (11-40) |
| ≥35 hours/week | 13 (5-30) | 23 (11-41) | 7 (3-17) | 21 (10-38) |
| Does not remember | 23 (8-49) | 30 (14-53) | 21 (8-43) | 31 (14-55) |
| *Time spent at preschool between the ages of 2 and 3 years (n=2056)^c^* | | |  |  |
| Up to 15 hours/week | 11 (4-26) | 20 (10-35) | 8 (3-18) | 18 (9-34) |
| 15-34 hours/week | 12 (5-27) | 21 (11-35) | 8 (3-17) | 19 (10-34) |
| ≥35 hours/week | 15 (6-32) | 23 (13-39) | 9 (4-18) | 21 (11-36) |
| Does not remember | 31 (13-57) | 32 (16-53) | 23 (10-45) | 33 (17-56) |
| *Time spent at preschool between the ages of 3 and 6 years (n=2208)^d^* | | |  |  |
| Up to 15 hours/week | 13 (5-31) | 25 (12-43) | 8 (3-18) | 22 (11-41) |
| 15-34 hours/week | 12 (5-26) | 22 (12-37) | 9 (5-19) | 20 (10-34) |
| ≥35 hours/week | 17 (7-34) | 24 (13-39) | 12 (6-23) | 23 (13-39) |
| Does not remember | 27 (11-53) | 32 (16-54) | 25 (11-46) | 39 (20-62) |

SDQ = Strengths and Difficulties Questionnaire; CES-DC = Center for Epidemiological Studies Depression Scale for Children; CI = confidence intervals.

^a^Adjusted for maternal age at child’s birth, parental country of origin, education, income and employment in the child’s first year of life and the child’s gestational age, birth weight, being singleton and having a congenital malformation; some covariate categories were combined due to small cell counts.

^b^Analyses included study participants who started preschool between the ages 1-2 years.

^c^Analyses included study participants who started preschool before the age of 3 years.

^d^Analyses included study participants who had been at preschool.

**Table S2.** Associations between age when the child started preschool and high total strengths and difficulties score, before and after adjustment for potential explanatory variables assessed at age 13

| **Potential explanatory variables** | **N^a^** | **The age when the child started preschool^b^** | | | |
| --- | --- | --- | --- | --- | --- |
|  |  | **12-15 months** | | **16-19 months** | |
|  |  | **OR (95 % CI) in the base model^c^** | **OR (95 % CI) in the base model^c^ + potential explanatory factor** | **OR (95 % CI) in the base model^c^** | **OR (95 % CI) in the base model^c^ + potential explanatory factor** |
| Maternal warmth as rated by the child | 1716 | 1.37 (1.00-1.87) | 1.32 (0.96-1.81) | 1.10 (0.82-1.48) | 1.09 (0.81-1.48) |
| Paternal warmth as rated by the child | 1715 | 1.39 (1.02-1.90) | 1.31 (0.95-1.81) | 1.10 (0.81-1.48) | 1.10 (0.81-1.49) |
| Parental warmth as rated by the parent | 1719 | 1.39 (1.02-1.90) | 1.39 (1.02-1.90) | 1.10 (0.82-1.49) | 1.10 (0.82-1.49) |
| Confidence in others | 1718 | 1.39 (1.02-1.90) | 1.37 (1.00-1.89) | 1.10 (0.82-1.48) | 1.14 (0.84-1.54) |
| Number of male friends | 1691 | 1.35 (0.99-1.85) | 1.37 (1.00-1.88) | 1.08 (0.80-1.46) | 1.10 (0.81-1.48) |
| Number of female friends | 1686 | 1.38 (1.01-1.89) | 1.38 (1.01-1.90) | 1.10 (0.82-1.49) | 1.11 (0.82-1.50) |
| Family support in school-related issues | 1718 | 1.38 (1.01-1.88) | 1.29 (0.94-1.79) | 1.10 (0.82-1.49) | 1.12 (0.82-1.53) |
| Academic results | 1598 | 1.34 (0.96-1.86) | 1.33 (0.95-1.85) | 1.11 (0.81-1.51) | 1.15 (0.84-1.58) |

OR = odds ratio; CI = confidence intervals.

^a^The number of participants without missing data on exposure, outcome and covariates.

^b^The age ≥20 months was considered as the reference.

^c^Adjusted for maternal age at child’s birth, parental country of origin, education, income and employment in the child’s first year of life and the child’s gestational age, birth weight, being singleton and having a congenital malformation.

**Table S3** Odds ratios and 95% confidence intervals for the association between preschool-related variables and mental health in propensity score matched analyses^a^

| **Type of exposure** | **OR (95% CI)** | | | |
| --- | --- | --- | --- | --- |
|  | **SDQ total score** | **SDQ internalizing problems** | **SDQ externalizing problems** | **Depression measured by CES-DC** |
| ***The age when the child started preschool^b^*** |  |  |  |  |
| 12-15 months | 1.34 (0.98-1.82) | 1.06 (0.82-1.38) | 1.50 (1.07-2.10) | 1.01 (0.76-1.34) |
| 16-19 months | 1.18 (0.86-1.61) | 1.04 (0.79-1.37) | 1.11 (0.77-1.61) | 0.96 (0.72-1.28) |
| ≥20 months | 1.00 | 1.00 | 1.00 | 1.00 |
| ***Time spent at preschool in different ages (hours/week)*** | |  |  |  |
| *Time spent at preschool between the ages of 1 and 2 years^c^* | |  |  |  |
| Up to 15 hours/week | 1.00 | 1.00 | 1.00 | 1.00 |
| 15-34 hours/week | 1.30 (0.78-2.14) | 0.91 (0.60-1.38) | 0.87 (0.51-1.47) | 1.11 (0.71-1.76) |
| ≥35 hours/week | 1.00 (0.55-1.81) | 0.94 (0.57-1.54) | 0.47 (0.25-0.87) | 0.97 (0.57-1.62) |
| Does not remember | 2.83 (1.12-7.19) | 1.78 (0.79-4.02) | 2.25 (0.98-5.17) | 2.71 (1.14-6.46) |
| *Time spent at preschool between the ages of 2 and 3 years^d^* | |  |  |  |
| Up to 15 hours/week | 1.00 | 1.00 | 1.00 | 1.00 |
| 15-34 hours/week | 0.66 (0.39-1.11) | 0.87 (0.57-1.33) | 0.59 (0.32-1.07) | 0.88 (0.57-1.37) |
| ≥35 hours/week | 1.12 (0.70-1.79) | 1.14 (0.76-1.72) | 0.79 (0.45-1.37) | 1.13 (0.73-1.74) |
| Does not remember | 2.44 (1.13-5.31) | 1.29 (0.64-2.59) | 3.40 (1.25-9.22) | 1.42 (0.68-2.97) |
| *Time spent at preschool between the ages of 3 and 6 years^e^* | |  |  |  |
| Up to 15 hours/week | 1.00 | 1.00 | 1.00 | 1.00 |
| 15-34 hours/week | 0.71 (0.34-1.48) | 0.80 (0.41-1.54) | 0.70 (0.27-1.84) | 0.85 (0.45-1.62) |
| ≥35 hours/week | 1.00 (0.51-1.96) | 0.56 (0.29-1.08) | 0.91 (0.39-2.14) | 0.68 (0.35-1.31) |
| Does not remember | 2.83 (1.12-7.19) | 1.89 (0.84-4.24) | 8.00 (1.84-34.79) | 2.50 (1.10-5.68) |

SDQ = Strengths and Difficulties Questionnaire; CES-DC = Center for Epidemiological Studies Depression Scale for Children; OR = odds ratio; CI = confidence intervals.

^a^Propensity scores were estimated using the following covariates: maternal age at the child’s birth, parental country of origin, education, income and employment in the child’s first year of life and the child’s gestational age, birth weight, being singleton and having a congenital malformation.

^b^Separate pairwise 1:1 propensity score matching analyses were performed for each exposure category with “≥20 months” as the reference group; the matched sample sizes were n =952 for 12-15 months and n =1022 for 16-19 months.

^c^Separate pairwise 1:1 propensity score-matched analyses were performed for each exposure category with “Up to 15 hours/week” as the reference group; the matched sample sizes were n =404 for “15–34 hours/week months”, n =366 for “≥35 hours/week” and n=122 for “Does not remember”.

^d^Separate pairwise 1:1 propensity score-matched analyses were performed for each exposure category with “Up to 15 hours/week” as the reference group; the matched sample sizes were n =436 for “15–34 hours/week months”, n =434 for “≥35 hours/week” and n=108 for “Does not remember”.

^e^Separate pairwise 1:1 propensity score-matched analyses were performed for each exposure category with “Up to 15 hours/week” as the reference group; the matched sample sizes were n =186 for “15–34 hours/week months”, n =186 for “≥35 hours/week” and n=100 for “Does not remember”.

**Table S4** Odds ratios and 95% confidence intervals for the association between age at start of preschool modeled as a continuous variable and mental health

| **Exposure** | **Adjusted OR (95% CI)^a^** | | | |
| --- | --- | --- | --- | --- |
|  | **SDQ total score** | **SDQ internalizing problems** | **SDQ externalizing problems** | **Depression measured by the CES-DC** |
| Age when the child started preschool, in month | 0.99 (0.98-1.01) | 0.99 (0.98-1.00) | 0.99 (0.98-1.01) | 1.00 (0.99-1.02) |

SDQ = Strengths and Difficulties Questionnaire; CES-DC = Center for Epidemiological Studies Depression Scale for Children; OR = odds ratio; CI = confidence intervals.

^a^Adjusted for maternal age at child’s birth, parental country of origin, education, income and employment in the child’s first year of life and the child’s gestational age, birth weight, being singleton and having a congenital malformation; analyses were restricted to participants with complete data on the exposure, outcome and adjusted covariates.

**Table S5** Odds ratios and 95% confidence intervals for the association between age at preschool entry dichotomized at 18 months and mental health

| **Age when the child started preschool** | **OR (95% CI)^a^** | | | |
| --- | --- | --- | --- | --- |
|  | **SDQ total score** | **SDQ internalizing problems** | **SDQ externalizing problems** | **Depression measured by CES-DC** |
| 12-17 months | 1.23 (0.96-1.57) | 0.92 (0.74-1.14) | 1.41 (1.07-1.86) | 1.01 (0.81-1.27) |
| ≥18 months | 1.00 | 1.00 | 1.00 | 1.00 |

SDQ = Strengths and Difficulties Questionnaire; CES-DC = Center for Epidemiological Studies Depression Scale for Children; OR = odds ratio; CI = confidence interval.

^a^Adjusted for maternal age at child’s birth, parental country of origin, education, income and employment in the child’s first year of life and the child’s gestational age, birth weight, being singleton and having a congenital malformation; analyses were restricted to participants with complete data on the exposure, outcome and covariates.

**Table S6** Least square means and 95% confidence intervals for scores on the Strengths and Difficulties Questionnaire and on the Center for Epidemiological Studies Depression Scale for Children according to preschool-related variables

| **Type of exposure** | **SDQ total score** | | **SDQ internalizing problems** | | **SDQ externalizing problems** | | **Depression measured by CES-DC** | |
| --- | --- | --- | --- | --- | --- | --- | --- | --- |
|  | **LS Mean (95% CI)^a^** | **P- value** | **LS Mean (95% CI)^a^** | **P- value** | **LS Mean (95% CI)^a^** | **P- value** | **LS Mean (95% CI)^a^** | **P-value** |
| ***The age when the child started preschool (n=2261)*** | |  |  |  |  |  |  |  |
| 12-15 months | 13 (11-15) | 0.03 | 7 (6-8) | 0.53 | 7 (6-8) | 0.02 | 21 (17-24) | 0.50 |
| 16-19 months | 12 (11-14) | 0.77 | 7 (6-8) | 0.99 | 6 (5-7) | 0.61 | 20 (17-24) | 0.84 |
| ≥20 months | 12 (11-14) | - | 7 (6-8) | - | 6 (5-7) | - | 20 (17-24) | - |
| ***Time spent at preschool in different ages (hours/week)*** | |  |  |  |  |  |  |  |
| *Time spent at preschool between the ages of 1 and 2 years (n=1779)^b^* | | |  |  |  |  |  |  |
| Up to 15 hours/week | 13 (11-15) | - | 7 (5-8) | - | 7 (6-8) | - | 20 (16-24) | - |
| 15-34 hours/week | 12 (10-14) | 0.33 | 6 (5-8) | 0.37 | 7 (5-8) | 0.36 | 20 (15-24) | 0.68 |
| ≥35 hours/week | 13 (11-15) | 0.68 | 7 (5-8) | 0.87 | 7 (5-8) | 0.42 | 20 (16-24) | 0.90 |
| Does not remember | 15 (12-17) | 0.01 | 8 (6-9) | 0.05 | 8 (7-9) | 0.03 | 23 (18-28) | 0.05 |
| *Time spent at preschool between the ages of 2 and 3 years (n=2056)^c^* | | |  |  |  |  |  |  |
| Up to 15 hours/week | 12 (10-14) | - | 6 (5-7) | - | 6 (5-7) | - | 19 (16-23) | - |
| 15-34 hours/week | 12 (11-14) | 0.45 | 6 (5-7) | 0.67 | 6 (5-7) | 0.77 | 20 (17-23) | 0.40 |
| ≥35 hours/week | 13 (11-14) | 0.06 | 7 (6-8) | 0.22 | 7 (6-8) | 0.18 | 20 (17-24) | 0.22 |
| Does not remember | 15 (13-17) | 0.00 | 8 (6-9) | 0.01 | 8 (7-9) | 0.00 | 25 (21-30) | 0.00 |
| *Time spent at preschool between the ages of 3 and 6 years (n=2208)^d^* | | |  |  |  |  |  |  |
| Up to 15 hours/week | 12 (10-14) | - | 6 (5-7) | - | 6 (5-8) | - | 20 (16-24) | - |
| 15-34 hours/week | 12 (10-13) | 0.56 | 6 (5-7) | 0.91 | 6 (5-7) | 0.86 | 19 (15-22) | 0.29 |
| ≥35 hours/week | 13 (11-14) | 0.27 | 7 (6-8) | 0.29 | 7 (6-8) | 0.17 | 21 (17-24) | 0.83 |
| Does not remember | 15 (12-17) | 0.01 | 7 (6-9) | 0.03 | 8 (7-9) | 0.01 | 25 (21-30) | 0.01 |

SDQ = Strengths and Difficulties Questionnaire; CES-DC = Center for Epidemiological Studies Depression Scale for Children; LS Mean = least square mean; CI = confidence interval.

^a^Adjusted for maternal age at child’s birth, parental country of origin, education, income and employment in the child’s first year of life and the child’s gestational age, birth weight, being singleton and having a congenital malformation; analyses were restricted to participants with complete data on the exposure, outcome and adjusted covariates.

^b^Analyses included study participants who started preschool between the ages 1-2 years.

^c^Analyses included study participants who started preschool before the age of 3 years.

^d^Analyses included study participants who had been at preschool.

**Table S7** Odds ratios and 95% confidence intervals for the association between preschool-related variables and mental health after multiple imputation for the covariates adjusted in the main model^a^

| **Type of exposure** | **Adjusted OR (95% CI)^b^** | | | |
| --- | --- | --- | --- | --- |
|  | **SDQ total score** | **SDQ internalizing problems** | **SDQ externalizing problems** | **Depression measured by the CES-DC** |
| ***The age when the child started preschool (n=2261)*** | |  |  |  |
| 12-15 months | 1.36 (1.04-1.77) | 1.05 (0.83-1.33) | 1.45 (1.08-1.96) | 1.04 (0.82-1.33) |
| 16-19 months | 1.07 (0.83-1.39) | 1.03 (0.82-1.28) | 0.97 (0.72-1.31) | 0.90 (0.72-1.14) |
| ≥20 months | 1.00 | 1.00 | 1.00 | 1.00 |
| ***Time spent at preschool in different ages (hours/week)*** | | |  |  |
| *Time spent at preschool between the ages of 1 and 2 years (n=1779)^c^* | | |  |  |
| Up to 15 hours/week | 1.00 | 1.00 | 1.00 | 1.00 |
| 15-34 hours/week | 1.07 (0.76-1.52) | 0.89 (0.66-1.19) | 0.87 (0.60-1.26) | 0.94 (0.69-1.29) |
| ≥35 hours/week | 1.16 (0.76-1.76) | 0.88 (0.61-1.26) | 0.88 (0.55-1.40) | 0.87 (0.60-1.28) |
| Does not remember | 1.77 (1.02-3.07) | 1.35 (0.82-2.23) | 1.89 (1.07-3.35) | 1.51 (0.91-2.52) |
| *Time spent at preschool between the ages of 2 and 3 years (n=2056)^d^* | | |  |  |
| Up to 15 hours/week | 1.00 | 1.00 | 1.00 | 1.00 |
| 15-34 hours/week | 1.10 (0.78-1.56) | 1.21 (0.89-1.63) | 0.84 (0.58-1.22) | 1.13 (0.82-1.55) |
| ≥35 hours/week | 1.41 (0.96-2.08) | 1.29 (0.92-1.82) | 1.14 (0.76-1.73) | 1.25 (0.88-1.77) |
| Does not remember | 2.70 (1.53-4.74) | 1.93 (1.13-3.29) | 2.35 (1.30-4.22) | 1.76 (1.01-3.05) |
| *Time spent at preschool between the ages of 3 and 6 years (n=2208)^e^* | | |  |  |
| Up to 15 hours/week | 1.00 | 1.00 | 1.00 | 1.00 |
| 15-34 hours/week | 1.02 (0.62-1.68) | 0.82 (0.55-1.24) | 1.29 (0.71-2.33) | 0.96 (0.62-1.48) |
| ≥35 hours/week | 1.40 (0.85-2.31) | 0.90 (0.59-1.36) | 1.73 (0.95-3.16) | 1.07 (0.68-1.68) |
| Does not remember | 2.24 (1.14-4.41) | 1.37 (0.75-2.52) | 3.34 (1.57-7.13) | 1.79 (0.95-3.34) |

SDQ = Strengths and Difficulties Questionnaire; CES-DC = Center for Epidemiological Studies Depression Scale for Children; OR = odds ratio; CI = confidence intervals.

^a^Multiple imputation was made for maternal age at child’s birth, parental country of origin, education, income and employment in the child’s first year of life and the child’s gestational age, birth weight, being singleton and having a congenital malformation.

^b^Adjusted for maternal age at child’s birth, parental country of origin, education, income and employment in the child’s first year of life and the child’s gestational age, birth weight, being singleton and having a congenital malformation.

^c^Analyses included study participants who started preschool between the ages 1-2 years.

^d^Analyses included study participants who started preschool before the age of 3 years.

^e^Analyses included study participants who had been at preschool.

**References**

1. Trost K, Biesecker G, Stattin H, et al. Not wanting parents' involvement: Sign of autonomy or sign of problems? *European Journal of Developmental Psychology* 2007;4:314-31.
