# Supplementary material for: Preschool care early in life and mental health in adolescence in Sweden: a cohort study
Source: BMJ Open. 2026 Apr 17;16(4):e105111. doi: 10.1136/bmjopen-2025-105111 (PMC13110637; doi:10.1136/bmjopen-2025-105111)
Supplement: Supplementary file 2 [file bmjopen-2025-105111.reviewer_comments.pdf]

PEER REVIEW HISTORY

BMJ Open publishes all reviews undertaken for accepted manuscripts. Reviewers are asked to complete a checklist review form and are provided with free text boxes to elaborate on their assessment. These free text comments are reproduced below.

ARTICLE DETAILS

Title (Provisional)

Preschool care early in life and mental health in adolescence in Sweden: a cohort study

Authors

Laszlo, Krisztina; Chen, Hua; Andersson, Filip; Galanti, Maria R

VERSION 1 - REVIEW

**Reviewer**	**1**
**Name**	**bouliotis, george**
**Affiliation**	**University of Warwick Warwick Clinical Trials Unit, Warwick Medical School**
**Date**	**28-Aug-2025**
**COI**	**None**

This is a very interesting research about the effect of preschool (early) care on mental health status during adolescence. Due to limited and often contradictory evidence, concrete research in this area is urgently needed. I encourage the authors to carry on researching this field whatever the outcome of this review.

The authors did access and developed a relevant database with a good potential to answer the research question(s). In their manuscript, they claimed that a statistically significant association was found, indicating that early schooling has a detrimental effect on mental health, thus, public health policy should take this finding into account.

However, their claims read like causal conclusions but in this case, they come from an observational study where assignment to groups is not at random. Although, I agree that if the finding is real, should be taking into consideration, I would expect to see additional evidence from the analysis implementing more robust methodology.

I strongly encourage the authors to fully address the list of comments below, in order to provide robust evidence from the sensitivity analyses that are needed here.

#Generic comments

1. The authors list a number of limitations, however they do not discuss the imbalance of the variables listed in the table1 across the groups. Despite that they do not provide p-values, it is obvious that significant differences do exist and potentially influenced the result (selection bias).

2. The authors report analyses of two mental health questionnaires with well-established scores of continuous nature using categorical (binary) splitting in order to focus only on mental ill-health. This is quite unconventional decision that is based on MCID thresholds, provided in references (Anna Goodman) for different research and questions designs. For assessing effects or changes in this type of (scored) questionnaires, the full score should be added here for each group (early, moderate, late), in addition to categorical thresholds (Logistic regression)

3. The analysis split the care effect sometimes in three and other times in two groups (early and late start timing). Only the early group indicates a statistical significance. I think the split in two groups only is preferable, given the low statistical power for some key confounders. In this case, it is of importance to additionally report the marginal probabilities by directly comparing the two groups in order to get a better idea of the effect of the timing on mental health.

4.As this is a typical observational research design, the (quite strong) claim "Conclusion: Starting preschool at very young age is associated with poor mental health in adolescence, particularly externalizing problems." should be accompanied by the following sensitivity analyses:

(i) Propensity Score Matching (PSM) analysis in order to eliminate group imbalance issues. For instance, check doi: https://doi.org/10.1080/09286586.2025.2548001 and doi: 10.1136/bmjopen-2015-008774

(ii) Report predicted probabilities (margins) for the effect per group

(iii) Provide in an appendix diagnostic checks of the model assumptions for the logistic regressions you have used, with emphasis on multicolinearity and influential values.

#Specific comments (page , raw)

p.1 Title: Do you mean "The effect of early preschool care on mental health in adolescence in Sweden. Results from the KUPOL longitudinal cohort"?

p.3 r.15 Design should also include data sources

p.3 r.17 Merge "Settings" with "Participants" and add more info

p.3 r.36 The statement emphasises on risk but then an Odds Ratio is reported. Although, this is not wrong it encourages wrong interpretation of Odds Ratios like Risk Ratios and that is wrong. The same applies to the whole manuscript. You need to consider either reporting Risk Ratios instead or alter the interpretation to focus on odds rather than plain risk.

p.3 r.49 The conclusion statement reads very strong like an unambiguous public health call. Given the limited evidence from the reported p-values only in this analysis, the strong tone here is not justified. Try rephrasing or provide more robust evidence.

p.3 r.57 Consider replacing "prevalence" with "frequency"

p.3 r.57-8 On what basis poor mental health in Sweden is justified. The statement reads problematic.

p.4 r.10 Remove "amount"

p.4 r.10 What means "with several dimensions"? Rephrase or remove

I would expect equal number of strength and limitation points

p.5 r.27 The term "Western" is outdated, do you mean "high income" countries or developed?

p.6 r.19 Do you mean "generously" subsidized?

p.6 r.24 Do you mean "unique" around the world

p.7 r.31-32 The statement reads weak and vague. You need to briefly but clearly state the research hypotheses

p.7 r.48 If the total population of the 541 schools is available, it should be added for transparency

"Study Population" The section repeats the word "study" in an unclear and misleading way. Do you mean that "longitudinal cohort study" is a "longitudinal electronic database" with various waves? The 3935 guardians gave consent to join your study or the KUPOL database? Please clarify and divide the section to "Data Source" and "(your) Study Population"

p.8 r.22 Replace "Measures" to read "Outcomes and Risk factors". Define and describe the study outcomes and the corresponding research questions. Explain the corresponding variables. Explain why all three are needed. Explain who collected SDQ and CES scales and how (postal, electronic?)

"Statistical Analysis"

The section needs careful re-writing with support from a qualified statistician as it reads vague at least. Analyses should match the study hypotheses, reported earlier and should be very specific and transparently presented

p.8 r.52 The introductory sentence reads weak and vague.

p.8 r.54 The sentence should be removed and explained in the previous section (Outcomes and Risk Factors)

p.9 r.3 I do not advocate unadjusted analyses, especially within the public health perspective. Do not report unadjusted results in the main paper and tables.

p.9 r.10 Listwise deletion almost always increases bias. You need to check and discuss this. Why multiple imputation was not undertaken?

p.9 r.12-31 The paragraph needs adequate discussion of the model(s) used and all adjusting variables in each model and the reasoning behind them. You should better consider report Risk Ratios (a GLM with log-link function instead of the logit-link function) as well as marginal probabilities that can quantify the risk appropriately and meaningfully.

p.9 r.38 A CONSORT-type diagram about the detailed identification of the study sample should be provided with justification about the mismatch between 3959 consented guardians and 2261 analysable(?).

p9. r.47 Table1 misses p-values as this is not a randomised study

p.9-10 The findings need to be verified with sensitivity analyses and the results should be properly reported in tables that mirror the current table1. In other words, tables should follow the 3 group mode as in the current table1

p.10 r.54-

The discussion (initial) subsection reads unreasonably strong given the inherent bias of the study. The remaining part is fine.

Th list of limitations should also include no use of proper group-balancing methods such as PSM and/or IPW for causal inference through eliminating selection bias

p.14 r.3 Unclear what the call for prospective studies means here, as in public health topics like this, prospective and efficient designs of this scale are almost never feasible. For this reason, the Real World Evidence methodology has been developed for the last 20 years in order to allow causal inference.

p.14 Ethics approval dated and numbered should be add somewhere

main Tables: They read unconventional and cluttered

T1: column1 should be titled as "characteristics". Remove "exposure". Should have four columns as group1,2,3 and then total and then p-values

The 3-group frequencies should exact match the total. This is not the case here.

T2: The table should be restructured to follow the previous table format. Then, risk estimated and 95% CIs should be carefully reported for the adjusted models only, and in a transparent way. Missing values should also be reported clearly. All models should be fully adjusted of all available covariates. All covariates should be stated at the "Methods" section once. Overall SDQ scores per group should also be provided in the table.

T3: The table should follow the same structure as tables 1 and 2. Transparent reporting of the estimates should be provided

Supplement Tables

Overall, the tables follow the expected structure reporting estimates by group but almost all of them are of minor or no importance for a public health reader. One or two maximum could be available in a supplement. Why the group m=20 is the reference group?

S1: Report only full-model estimates. Is the "N" column for total frequencies? How that "total" applies to both groups in the table? Personally, I like the no-reporting of any p-values here.

S2 and S3: The table reports some large ORs with corresponding confidence intervals that does not include 1 (e.g. Girl) but there is a p-value next to them that comes from the interaction term. This is a quite misleading display. Please try a better display for those results, if of importance. Textual only reporting somewhere in the Results section should be preferable

S4 and S5: Heavily cluttered tables with loads of estimates with p-values here and there. I cannot see where those tables are discussed in the manuscript.

Supplement Information

The section is useful but too lengthy and should be truncated by 15% at least

---

**Reviewer**	**2**
**Name**	**Högberg, Björn**
**Affiliation**	**Umea Universitet, Social Work**
**Date**	**18-Nov-2025**
**COI**	**None**

The paper addresses a substantively important topic, which is commendable. The topic is also politically controversial, which should by no means discourage scholars from studying it, but it does mean that extra caution may be required in interpreting the results.

My main objection is that the paper, like many papers in public health and the social sciences, wavers between being causal and associational/descriptive in focus. The language is clearly associational, and causal terms are carefully avoided when describing the results. However, the research question is undoubtedly motivated from a causal perspective: an association between childcare and mental health is not very interesting in itself. Moreover, the introduction and discussion are largely devoted to topics that implicitly assume causal relationships (discussion of policy in the intro, mechanisms on p 11). Finally, the authors adjust for several (I think nine) confounders, and covariate/confounder adjustment is typically motivated by reducing bias (and hence to capture identify effects; however, the authors do not explicitly motivate why they include covariates in this study).

What I am getting at is that I think that the paper needs a more elaborate discussion of causality and confounding/bias. This is not merely something that should only be mentioned as a limitation; readers deserve to know to what extent the odds ratios can be given a causal interpretation, and what the main sources of bias may be. In particular, I think it would be highly useful with an elaborate discussion concerning selection into preschool and especially early preschool. Who are the parents who decide to put their child into preschool at an early age? What are their reasons for doing so? I can imagine that parents are more prone to send their child to preschool early if they have had a difficult first year, either because the child is "turbulent", cries a lot or is hyperactive or because the parents have had difficulties and are emotionally exhausted (e.g., maternal depression). I can also imagine that parents with financial difficulties are less likely to stay at home with the child for a long time because they cannot afford to do so. Finally, I can imagine that parents with progressive parenting values are more likely to send their child to preschool early compared to parents with more conservative values, and the progressive parents may also be more tolerant of externalizing behaviors and/or more open about discussing internalizing problems with the child. These are just some ideas: the point is that the authors should carefully think through what kind of social processes that they are trying to capture, and also guide the readers in this context. The authors can actually partly investigate this themselves using their data. What differences do they observe across exposure groups, and what do these differences tell them about possible sources of bias?

A second major concern: The stated aim is to investigate "whether age at starting preschool and weekly hours spent at preschool in different ages are associated with mental ill-health". This is an omnibus aim (or possible two aims: one for each exposure) that is tested using four different outcome variables and different types of exposure variables. In total, tables 2 and 3 include 32 odds ratios that may be said to investigate this aim (I here count the adjusted odds ratios for the different categories of age of starting and time at preschool for the four outcomes, and exclude the unadjusted odds ratios and the "does not remember" category). 2 of these 32 odds ratios are statistically significant, and in both cases the CI is quite close to overlapping with 1. Strictly speaking, the authors should probably apply multiple testing correction given the large number of tests of the same research question (or implicit null hypothesis). A standard Bonferroni correction would then render both significant effects non-significant. Now, multiple testing correction is partly a philosophical issue, and something I do not always practice myself. The main point is that the results can be interpreted in two radically different ways: (1) as showing that early preschool starting age is associated with (some) mental health problems, based on the two significant odds ratios, or (2) as showing that the evidence for an association between preschool attendance (starting age and hour/week) and mental health is fairly weak, fragile, and model dependent; the second interpretation also accounts for the 30 or so non-significant odds ratios. My reading of the results is that the second interpretation is more reasonable and better aligned with the combined evidence presented in tables 2 and 3. I also think that the

second interpretation is supported by the rather weak effects of the two significant odds ratios (both having CIs almost overlapping with 1, meaning that a small amount of unobserved confounding would render them nonsignificant) and by the inconsistencies of the results: If early preschool attendance truly causes mental health problems, then we should expect to observe a dose-response relationship between number of hours in age 1-2 and mental health problems, but we don't in this case (the effects of hours are non-significant, and the point estimates are actually in the reverse direction for three out of four outcomes). The meaning of these inconsistencies should also be discussed in the paper.

A partly related comment concerns the sample. The authors touch upon the non-response rate and representativeness of the sample in the limitations-section. However, I think this is potentially a bigger issue that should be introduced already on p 6 ("Study population"). 541 schools were sampled, 101 of which participated. That's a school-level response-rate of less than 20%. Of the 12 512 students in these schools, 3 959 provided consent (or rather: their guardians did), which is 32 %. Of the 3 959 who consented, 2 563 provided data on mental health, and somewhat fewer also on the pre-school variables. Then we are down at an individual-level response rate of around 20%. Assuming that the number of students does not vary between responding and non-responding schools (I have no idea if this is the case, but just for the sake of the argument), this gives a total response rate of 4% (0.2*0.2=0.04).

This final sample is probably selected, and possibly heavily so, in numerous ways. Such a response rate requires a careful discussion of representativeness, selection, bias and so on. Admittedly, such a discussion is partly provided in the limitations-section, but I feel that it perhaps deserves even greater attention (not merely as a limitation, but also concerning the implications).

Additionally, the authors have access to register data that can be linked to the survey. Is it possible to construct weights based on register data, such that groups with low response-rates are given a greater weight?

Minor comments

P 12: "Several earlier studies have suggested that there may be some benefit for enrolling early in a high quality daycare for children from disadvantaged as opposed to those from more advantaged families. It is therefore plausible that associations observed in our study may be underestimated, given the protective factors associated with a highly educated and Swedish-born population, i.e. categories that are overrepresented in our sample."

I don't follow how the second sentence builds on the first. If the type of children that gain from attending preschool early (the disadvantaged) are underrepresented, then the associations are more likely to be overestimated, not underestimated, right? Or am I missing something here?

P 7: "A detailed description of the outcomes and the covariates is provided in the Supplementary material." I agree that detailed descriptions can be included in the

supplement. However, readers of the main manuscript would gain from having some notion of what covariates are included, and why. Two or three sentences would suffice.

The paper is mostly well-written and easy to follow. There are some errors that could be picked up by a careful proof-reading, however. Some examples: P 9, line 12: “There was no an association between…”. Either it is “no” association, or “an” association? P 10, line 42: “A study by Hagekull and Bohlin”. “By” shouldn’t be in superscript.

P 2 of the supplement: “Information on paternal and maternal income and employment status during the year after childbirth …” Income and employment the year after childbirth may not be very meaningful considering that many parents are on parental leave then, and thus have lower incomes and possibly no employment. Moreover, these measures may increase rather than reduce bias since they are differentially associated with age of preschool attendance. Parents who send their children to preschool early will likely have higher incomes, all else being equal, because they can go back to work.

I may come about as too harsh here: I do commend the authors for studying a substantively important issue with important policy implications, which unfortunately is not always the case in public health research. I also think that the manuscript is well-written and the analyses are sound. I would like to see the manuscript published in some form. However, I’m not sure if I agree with the author’s main take-away of the paper, and I think causality should be addressed head-on (given the policy implications of the findings).

VERSION 1 - AUTHOR RESPONSE

Reviewer #1: Dr. George Bouliotis, University of Warwick Warwick Clinical Trials Unit

Comments to the Author:

This is a very interesting research about the effect of preschool (early) care on mental health status during adolescence. Due to limited and often contradictory evidence, concrete research in this area is urgently needed. I encourage the authors to carry on researching this field whatever the outcome of this review.

The authors did access and developed a relevant database with a good potential to answer the research question(s). In their manuscript, they claimed that a statistically significant association was found, indicating that early schooling has a detrimental effect on mental health, thus, public health policy should take this finding into account.

However, their claims read like causal conclusions but in this case, they come from an observational study where assignment to groups is not at random. Although, I agree that if the finding is real, should be taking into consideration, I would expect to see additional evidence from the analysis implementing more robust methodology.
I strongly encourage the authors to fully address the list of comments below, in order to provide robust evidence from the sensitivity analyses that are needed here.

Response:

Thank you for the positive comments on our study.

Comment 1:

#Generic comments

1. The authors list a number of limitations, however they do not discuss the imbalance of the variables listed in the table1 across the groups. Despite that they do not provide p-values, it is obvious that significant differences do exist and potentially influenced the result (selection bias).

Response:

We now compare the characteristics of study participants across categories of age at preschool entry by means of chi-square tests in case of categorical variables and by means of Kruskal-Wallis tests in case of the continuous variables with non-normal distribution. We added the corresponding p-values to Table 1. We have now performed Propensity Score Matching (PSM) analyses for all exposure-outcome associations to balance the exposure groups on the available

confounders. In addition, we discuss how known and unknown differences between exposure groups may have affected the results.

Please see the Statistical analysis section (page 9)

We compared exposure groups by means of chi-square tests in case of categorical variables and by means of Kruskal-Wallis tests in case of continuous variables with non-normal distribution.

Please see Table 1 (pages 21-25)

Table 1 *Characteristics of study participants according to age of enrolling in preschool*

Characteristics	Total (n=2261)	Age of the child when starting preschool (months)			
		12-15 (n=642)	16-19 (n=869)	≥20 (n=750)	P-value[a]
Categorical variables, n (%)					
Child's gender					0.99
Boy	1065 (47.1)	302 (47.0)	409 (47.1)	354 (47.2)	
Girl	1196 (52.9)	340 (53.0)	460 (52.9)	396 (52.8)	
Mother's age when the child was born (years)					<0.01
≤25	195 (8.6)	71 (11.1)	77 (8.9)	47 (6.3)	
26-30	719 (31.8)	196 (30.5)	315 (36.2)	208 (27.7)	
31-35	759 (33.6)	217 (33.8)	280 (32.2)	262 (34.9)	
36-40	315 (13.9)	78 (12.1)	115 (13.2)	122 (16.3)	
≥41	62 (2.7)	12 (1.9)	19 (2.2)	31 (4.1)	
Missing	211 (9.3)	68 (10.6)	63 (7.2)	80 (10.7)	
Both parents were born in Sweden					0.02
Yes	1736 (76.8)	466 (72.6)	688 (79.2)	582 (77.6)	
No	317 (14.0)	109 (17.0)	106 (12.2)	102 (13.6)	
Missing	208 (9.2)	67 (10.4)	75 (8.6)	66 (8.8)	
Paternal education level (years)					0.45
0-9	150 (6.6)	49 (7.6)	53 (6.1)	48 (6.4)	
10-14	1479 (65.4)	425 (66.2)	559 (64.3)	495 (66.0)	
≥15	621 (27.5)	166 (25.9)	256 (29.5)	199 (26.5)	
Missing	11 (0.5)	2 (0.3)	1 (0.1)	8 (1.1)	
Maternal educational level (years)					0.32
0-9	79 (3.5)	30 (4.7)	30 (3.5)	19 (2.5)	
10-14	1347 (59.6)	380 (59.2)	516 (59.4)	451 (60.1)	
≥15	828 (36.6)	231 (36.0)	322 (37.1)	275 (36.7)	
Missing	7 (0.3)	1 (0.2)	1 (0.1)	5 (0.7)	

Paternal employment status in the child's first year of life					*<0.01*
Employed	*2033 (89.9)*	*578 (90.0)*	*813 (93.6)*	*642 (85.6)*	
Unemployed	*126 (5.6)*	*41 (6.4)*	*28 (3.2)*	*57 (7.6)*	
Missing	*102 (4.5)*	*23 (3.6)*	*28 (3.2)*	*51 (6.8)*	
Maternal employment status in the child's first year of life					*0.49*
Employed	*1732 (76.6)*	*493 (76.8)*	*684 (78.7)*	*555 (74.0)*	
Unemployed	*435 (19.2)*	*126 (19.6)*	*159 (18.3)*	*150 (20.0)*	
Missing	*94 (4.2)*	*23 (3.6)*	*26 (3.0)*	*45 (6.0)*	
Paternal income in the child's first year of life					*0.02*
Low tertile	*710 (31.4)*	*213 (33.2)*	*243 (28.0)*	*254 (33.9)*	
Middle tertile	*728 (32.2)*	*214 (33.3)*	*297 (34.2)*	*217 (28.9)*	
High tertile	*721 (31.9)*	*192 (29.9)*	*301 (34.6)*	*228 (30.4)*	
Missing	*102 (4.5)*	*23 (3.6)*	*28 (3.2)*	*51 (6.8)*	
Maternal income in the child's first year of life					*<0.01*
Low tertile	*712 (31.5)*	*221 (34.4)*	*234 (26.9)*	*257 (34.3)*	
Middle tertile	*737 (32.6)*	*211 (32.9)*	*308 (35.4)*	*218 (29.1)*	
High tertile	*718 (31.8)*	*187 (29.1)*	*301 (34.6)*	*230 (30.7)*	
Missing	*94 (4.2)*	*23 (3.6)*	*26 (3.0)*	*45 (6.0)*	
Gestational age (weeks)					*<0.01*
<32	*12 (0.5)*	*0*	*5 (0.6)*	*7 (0.9)*	
32-36	*106 (4.7)*	*22 (3.4)*	*34 (3.9)*	*50 (6.7)*	
≥37	*1953 (86.4)*	*575 (89.6)*	*771 (88.7)*	*607 (80.9)*	
Missing	*190 (8.4)*	*45 (7.0)*	*59 (6.8)*	*86 (11.5)*	
Birth weight (gram)					*0.01*
<2500	*74 (3.3)*	*13 (2.0)*	*26 (3.0)*	*35 (4.7)*	
≥2500	*1994 (88.2)*	*581 (90.5)*	*784 (90.2)*	*629 (83.9)*	
Missing	*193 (8.5)*	*48 (7.5)*	*59 (6.8)*	*86 (11.5)*	
Singleton					*<0.01*
No	*75 (3.3)*	*12 (1.9)*	*24 (2.8)*	*39 (5.2)*	
Yes	*2186 (96.7)*	*630 (98.1)*	*845 (97.2)*	*711 (94.8)*	
Congenital malformation					*0.02*
Yes	*65 (2.9)*	*28 (4.4)*	*18 (2.1)*	*19 (2.5)*	
No	*2196 (97.1)*	*614 (95.6)*	*851 (97.9)*	*731 (97.5)*	
Number of male friends					*0.35*
0	*302 (13.4)*	*80 (12.5)*	*110 (12.7)*	*112 (14.9)*	
1	*249 (11.0)*	*61 (9.5)*	*109 (12.5)*	*79 (10.5)*	
2	*314 (13.9)*	*93 (14.5)*	*122 (14.0)*	*99 (13.2)*	
≥3	*1352 (59.8)*	*399 (62.1)*	*513 (59.0)*	*440 (58.7)*	
Missing	*44 (1.9)*	*9 (1.4)*	*15 (1.7)*	*20 (2.7)*	
Number of female friends					*0.83*
0	*215 (9.5)*	*62 (9.7)*	*87 (10.0)*	*66 (8.8)*	
1	*197 (8.7)*	*52 (8.1)*	*77 (8.9)*	*68 (9.1)*	
2	*317 (14.0)*	*82 (12.8)*	*129 (14.8)*	*106 (14.1)*	
≥3	*1481 (65.5)*	*433 (67.4)*	*557 (64.1)*	*491 (65.5)*	
Missing	*51 (2.3)*	*13 (2.0)*	*19 (2.2)*	*19 (2.5)*	

Sum of grades in Swedish, English and Mathematics (grade 7)					*0.02*
<35	*292 (12.9)*	*91 (14.2)*	*101 (11.6)*	*100 (13.3)*	
35-50	*1424 (63.0)*	*401 (62.5)*	*538 (61.9)*	*485 (64.7)*	
>50	*384 (17.0)*	*92 (14.3)*	*176 (20.3)*	*116 (15.5)*	
Missing	*161 (7.1)*	*58 (9.0)*	*54 (6.2)*	*49 (6.5)*	
Continuous variables, median					
Maternal warmth as rated by the child	*16*	*16*	*16*	*16*	*0.02*
Paternal warmth as rated by the child	*16*	*15*	*16*	*16*	*0.03*
Parental warmth as rated by the parent	*17*	*17*	*17*	*17*	*0.79*
Confidence in others	*7*	*7*	*8*	*8*	*0.01*
Family support in school-related issues	*20*	*19*	*20*	*20*	*0.06*

[a]P-values were estimated using chi-square tests for categorical variables and Kruskal-Wallis tests for continuous variables.

Please see the Statistical analysis section (page 10)

We performed several sensitivity analyses. First, to balance baseline characteristics between exposure groups, we run analyses with propensity score matching (PSM).

Please see Table S3 (Supplementary material)

Table S3 *Odds ratios and 95% confidence intervals for the association between preschool-related variables and mental health in propensity score matched analyses*[a]

Type of exposure	***OR (95% CI)***			
	SDQ total score	***SDQ internalizing problems***	***SDQ externalizing problems***	***Depression measured by CES-DC***
The age when the child started preschool[b]				
12-15 months	*1.34 (0.98-1.82)*	*1.06 (0.82-1.38)*	*1.50 (1.07-2.10)*	*1.01 (0.76-1.34)*
16-19 months	*1.18 (0.86-1.61)*	*1.04 (0.79-1.37)*	*1.11 (0.77-1.61)*	*0.96 (0.72-1.28)*
≥20 months	*1.00*	*1.00*	*1.00*	*1.00*
Time spent at preschool in different ages (hours/week)				
Time spent at preschool between the ages of 1 and 2 years[c]				
Up to 15 hours/week	*1.00*	*1.00*	*1.00*	*1.00*
15-34 hours/week	*1.30 (0.78-2.14)*	*0.91 (0.60-1.38)*	*0.87 (0.51-1.47)*	*1.11 (0.71-1.76)*
≥35 hours/week	*1.00 (0.55-1.81)*	*0.94 (0.57-1.54)*	*0.47 (0.25-0.87)*	*0.97 (0.57-1.62)*
Does not remember	*2.83 (1.12-7.19)*	*1.78 (0.79-4.02)*	*2.25 (0.98-5.17)*	*2.71 (1.14-6.46)*
Time spent at preschool between the ages of 2 and 3 years[d]				
Up to 15 hours/week	*1.00*	*1.00*	*1.00*	*1.00*
15-34 hours/week	*0.66 (0.39-1.11)*	*0.87 (0.57-1.33)*	*0.59 (0.32-1.07)*	*0.88 (0.57-1.37)*
≥35 hours/week	*1.12 (0.70-1.79)*	*1.14 (0.76-1.72)*	*0.79 (0.45-1.37)*	*1.13 (0.73-1.74)*
Does not remember	*2.44 (1.13-5.31)*	*1.29 (0.64-2.59)*	*3.40 (1.25-9.22)*	*1.42 (0.68-2.97)*
Time spent at preschool between the ages of 3 and 6 years[e]				
Up to 15 hours/week	*1.00*	*1.00*	*1.00*	*1.00*
15-34 hours/week	*0.71 (0.34-1.48)*	*0.80 (0.41-1.54)*	*0.70 (0.27-1.84)*	*0.85 (0.45-1.62)*
≥35 hours/week	*1.00 (0.51-1.96)*	*0.56 (0.29-1.08)*	*0.91 (0.39-2.14)*	*0.68 (0.35-1.31)*
Does not remember	*2.83 (1.12-7.19)*	*1.89 (0.84-4.24)*	*8.00 (1.84-34.79)*	*2.50 (1.10-5.68)*

SDQ = Strengths and Difficulties Questionnaire; CES-DC = Center for Epidemiological Studies Depression Scale for Children; OR = odds ratio; CI = confidence intervals.

[a] *Propensity scores were estimated using the following covariates: maternal age at the child's birth, parental country of origin, education, income and employment in the child's first year of life and the child's gestational age, birth weight, being singleton and having a congenital malformation.*
[b] *Separate pairwise 1:1 propensity score matching analyses were performed for each exposure category with "≥20 months" as the reference group; the matched sample sizes were n =952 for 12-15 months and n =1022 for 16-19 months.*
[c] *Separate pairwise 1:1 propensity score-matched analyses were performed for each exposure category with "Up to 15 hours/week" as the reference group; the matched sample sizes were n =404 for "15–34 hours/week months", n =366 for "≥35 hours/week" and n=122 for "Does not remember".*
[d] *Separate pairwise 1:1 propensity score-matched analyses were performed for each exposure category with "Up to 15 hours/week" as the reference group; the matched sample sizes were n =436 for "15–34 hours/week months", n =434 for "≥35 hours/week" and n=108 for "Does not remember".*
[e] *Separate pairwise 1:1 propensity score-matched analyses were performed for each exposure category with "Up to 15 hours/week" as the reference group; the matched sample sizes were n =186 for "15–34 hours/week months", n =186 for "≥35 hours/week" and n=100 for "Does not remember".*

Please see the Discussion section (pages 14-15)

Several methodological aspects need to be addressed when interpreting the observed associations in addition to the possibility for a causal explanation. The first one is selection. Parents who choose to enroll their children in daycare at early ages and their children may differ from their counterparts enrolling in daycare in later ages on personal characteristics and contextual factors that make it difficult to make predictions on the magnitude and directions of the potential bias. For example, some parents may have chosen an early enrolment in daycare because of the child's behavioral problems (e.g., frequent crying or being hyperactive), and/or because of the parent himself/herself had poor mental health. On the other hand, socially well integrated parents with progressive parenting values may send their children to daycare earlier than parents who do not endorse trust in the community services; the former may also be more tolerant to externalizing behaviours in the child. Despite of the rather comprehensive attempt to control for confounders we cannot exclude the possibility that the modest association between start of daycare at the age of 12-15 months and the odds of high SDQ and high externalizing problems in adolescence may be due to bias.

Comment 2:

2. The authors report analyses of two mental health questionnaires with well-established scores of continuous nature using categorical (binary) splitting in order to focus only on mental ill-health. This is quite unconventional decision that is based on MCID thresholds, provided in references (Anna Goodman) for different research and questions designs. For assessing effects or changes in this type of (scored) questionnaires, the full score should be added here for each group (early, moderate, late), in addition to categorical thresholds (Logistic regression)

Response:

We acknowledge that dichotomization of continuous outcomes may reduce statistical power and obscure more subtle variations in symptom levels; nevertheless, we chose these analyses as they may be informative with respect to clinical relevance. We have now included also analyses in which we treated the mental health questionnaire scores as continuous.

Please see the Statistical analysis section (page 10)

Third, we repeated our main analyses with the outcome treated as continuous variable; we used linear regression to generate and compare least square means.

Please see Table S6 (Supplementary material)

Table S6 *Least square means and 95% confidence intervals for scores on the Strengths and Difficulties Questionnaire and on the Center for Epidemiological Studies Depression Scale for Children according to preschool-related variables*

Type of exposure	***SDQ total score***		***SDQ internalizing problems***		***SDQ externalizing problems***		***Depression measured by CES-DC***	
	LS Mean (95% CI)[a]	***P-value***	***LS Mean (95% CI)[a]***	***P-value***	***LS Mean (95% CI)[a]***	***P-value***	***LS Mean (95% CI)[a]***	***P-value***
The age when the child started preschool (n=2261)								
12-15 months	*13 (11-15)*	*0.03*	*7 (6-8)*	*0.53*	*7 (6-8)*	*0.02*	*21 (17-24)*	*0.50*
16-19 months	*12 (11-14)*	*0.77*	*7 (6-8)*	*0.99*	*6 (5-7)*	*0.61*	*20 (17-24)*	*0.84*
≥20 months	*12 (11-14)*	*-*	*7 (6-8)*	*-*	*6 (5-7)*	*-*	*20 (17-24)*	*-*
Time spent at preschool in different ages (hours/week)								
Time spent at preschool between the ages of 1 and 2 years (n=1779)[b]								
Up to 15 hours/week	*13 (11-15)*	*-*	*7 (5-8)*	*-*	*7 (6-8)*	*-*	*20 (16-24)*	*-*
15-34 hours/week	*12 (10-14)*	*0.33*	*6 (5-8)*	*0.37*	*7 (5-8)*	*0.36*	*20 (15-24)*	*0.68*
≥35 hours/week	*13 (11-15)*	*0.68*	*7 (5-8)*	*0.87*	*7 (5-8)*	*0.42*	*20 (16-24)*	*0.90*
Does not remember	*15 (12-17)*	*0.01*	*8 (6-9)*	*0.05*	*8 (7-9)*	*0.03*	*23 (18-28)*	*0.05*
Time spent at preschool between the ages of 2 and 3 years (n=2056)[c]								
Up to 15 hours/week	*12 (10-14)*	*-*	*6 (5-7)*	*-*	*6 (5-7)*	*-*	*19 (16-23)*	*-*
15-34 hours/week	*12 (11-14)*	*0.45*	*6 (5-7)*	*0.67*	*6 (5-7)*	*0.77*	*20 (17-23)*	*0.40*
≥35 hours/week	*13 (11-14)*	*0.06*	*7 (6-8)*	*0.22*	*7 (6-8)*	*0.18*	*20 (17-24)*	*0.22*
Does not remember	*15 (13-17)*	*0.00*	*8 (6-9)*	*0.01*	*8 (7-9)*	*0.00*	*25 (21-30)*	*0.00*
Time spent at preschool between the ages of 3 and 6 years (n=2208)[d]								
Up to 15 hours/week	*12 (10-14)*	*-*	*6 (5-7)*	*-*	*6 (5-8)*	*-*	*20 (16-24)*	*-*
15-34 hours/week	*12 (10-13)*	*0.56*	*6 (5-7)*	*0.91*	*6 (5-7)*	*0.86*	*19 (15-22)*	*0.29*
≥35 hours/week	*13 (11-14)*	*0.27*	*7 (6-8)*	*0.29*	*7 (6-8)*	*0.17*	*21 (17-24)*	*0.83*
Does not remember	*15 (12-17)*	*0.01*	*7 (6-9)*	*0.03*	*8 (7-9)*	*0.01*	*25 (21-30)*	*0.01*

SDQ = Strengths and Difficulties Questionnaire; CES-DC = Center for Epidemiological Studies Depression Scale for Children; LS Mean = least square mean; CI = confidence interval.

[a]Adjusted for maternal age at child's birth, parental country of origin, education, income and employment in the child's first year of life and the child's gestational age, birth weight, being singleton and having a congenital malformation; analyses were restricted to participants with complete data on the exposure, outcome and adjusted covariates.
[b]Analyses included study participants who started preschool between the ages 1-2 years.
[c]Analyses included study participants who started preschool before the age of 3 years.
[d]Analyses included study participants who had been at preschool.

Comment 3:

3. The analysis split the care effect sometimes in three and other times in two groups (early and late start timing). Only the early group indicates a statistical significance. I think the split in two groups only is preferable, given the low statistical power for some key confounders. In this case, it is of importance to additionally report the marginal probabilities by directly comparing the two groups in order to get a better idea of the effect of the timing on mental health.

Response:

We would like to clarify that age at preschool entry was consistently categorized into three groups across all primary analyses. Specifically, age at start of preschool was categorized based on tertiles, which allowed us to consider non-linear effects and to distinguish the group of children with particularly early preschool entry from those starting at or after the more typical preschool entry age in this setting.

In response to the reviewer's suggestion, we conducted additional sensitivity analyses in which age at preschool entry was dichotomized at the median (18 months). In addition, we performed adjusted logistic regression analyses modeling age at preschool entry as a continuous exposure.

Please see the Statistical analysis section (page 10)

Second, to analyze whether our categorization of age at start of preschool influenced the results we repeated our main models with the variable treated as (1) continuous and (2) dichotomized at the median, i.e., at 18 months.

Please see Table S4 (Supplementary material)

***Table S4** Odds ratios and 95% confidence intervals for the association between age at start of preschool modeled as a continuous variable and mental health*

Exposure	***Adjusted OR (95% CI)[a]***			
	SDQ total score	***SDQ internalizing problems***	***SDQ externalizing problems***	***Depression measured by the CES-DC***
Age when the child started preschool, in month	*0.99 (0.98-1.01)*	*0.99 (0.98-1.00)*	*0.99 (0.98-1.01)*	*1.00 (0.99-1.02)*

SDQ = Strengths and Difficulties Questionnaire; CES-DC = Center for Epidemiological Studies Depression Scale for Children; OR = odds ratio; CI = confidence intervals.
[a]Adjusted for maternal age at child's birth, parental country of origin, education, income and employment in the child's first year of life and the child's gestational age, birth weight, being singleton and having a congenital malformation; analyses were restricted to participants with complete data on the exposure, outcome and adjusted covariates.

Please see Table S5 (Supplementary material)

***Table S5** Odds ratios and 95% confidence intervals for the association between age at preschool entry dichotomized at 18 months and mental health*

Age when the child started preschool	***OR (95% CI)[a]***			
	SDQ total score	***SDQ internalizing problems***	***SDQ externalizing problems***	***Depression measured by CES-DC***
12-17 months	*1.23 (0.96-1.57)*	*0.92 (0.74-1.14)*	*1.41 (1.07-1.86)*	*1.01 (0.81-1.27)*
≥18 months	*1.00*	*1.00*	*1.00*	*1.00*

SDQ = Strengths and Difficulties Questionnaire; CES-DC = Center for Epidemiological Studies Depression Scale for Children; OR = odds ratio; CI = confidence interval.
[a]Adjusted for maternal age at child's birth, parental country of origin, education, income and employment in the child's first year of life and the child's gestational age, birth weight, being singleton and having a congenital malformation; analyses were restricted to participants with complete data on the exposure, outcome and covariates.

Comment 4:

4.As this is a typical observational research design, the (quite strong) claim "Conclusion: Starting preschool at very young age is associated with poor mental health in adolescence, particularly externalizing problems." should be accompanied by the following sensitivity analyses:

(i) Propensity Score Matching (PSM) analysis in order to eliminate group imbalance issues. For instance, check doi: https://doi.org/10.1080/09286586.2025.2548001 and doi: 10.1136/bmjopen-2015-008774

(ii) Report predicted probabilities (margins) for the effect per group

(iii) Provide in an appendix diagnostic checks of the model assumptions for the logistic regressions you have used, with emphasis on multicolinearity and influential values.

Response:

In addition to our multivariate analyses adjusting for a large number of possible confounders, we have now performed also PSM analyses for all-exposure outcome associations to balance the exposure groups on the available confounders. We now also include the adjusted predicted probabilities (Table S1). We have also performed analyses for testing multicollinearity and influential values. Furthermore, we have rewritten the conclusion to tone down causal interpretations.

Please see the Statistical analysis section (page 10)

First, to balance baseline characteristics between exposure groups, we run analyses with propensity score matching (PSM).

Please see Table S3 (Supplementary material)

Table S3 *Odds ratios and 95% confidence intervals for the association between preschool-related variables and mental health in propensity score matched analyses*[a]

Type of exposure	***OR (95% CI)***			
	SDQ total score	***SDQ internalizing problems***	***SDQ externalizing problems***	***Depression measured by CES-DC***
The age when the child started preschool[b]				
12-15 months	*1.34 (0.98-1.82)*	*1.06 (0.82-1.38)*	*1.50 (1.07-2.10)*	*1.01 (0.76-1.34)*
16-19 months	*1.18 (0.86-1.61)*	*1.04 (0.79-1.37)*	*1.11 (0.77-1.61)*	*0.96 (0.72-1.28)*
≥20 months	*1.00*	*1.00*	*1.00*	*1.00*
Time spent at preschool in different ages (hours/week)				
Time spent at preschool between the ages of 1 and 2 years[c]				
Up to 15 hours/week	*1.00*	*1.00*	*1.00*	*1.00*
15-34 hours/week	*1.30 (0.78-2.14)*	*0.91 (0.60-1.38)*	*0.87 (0.51-1.47)*	*1.11 (0.71-1.76)*
≥35 hours/week	*1.00 (0.55-1.81)*	*0.94 (0.57-1.54)*	*0.47 (0.25-0.87)*	*0.97 (0.57-1.62)*
Does not remember	*2.83 (1.12-7.19)*	*1.78 (0.79-4.02)*	*2.25 (0.98-5.17)*	*2.71 (1.14-6.46)*
Time spent at preschool between the ages of 2 and 3 years[d]				
Up to 15 hours/week	*1.00*	*1.00*	*1.00*	*1.00*
15-34 hours/week	*0.66 (0.39-1.11)*	*0.87 (0.57-1.33)*	*0.59 (0.32-1.07)*	*0.88 (0.57-1.37)*
≥35 hours/week	*1.12 (0.70-1.79)*	*1.14 (0.76-1.72)*	*0.79 (0.45-1.37)*	*1.13 (0.73-1.74)*
Does not remember	*2.44 (1.13-5.31)*	*1.29 (0.64-2.59)*	*3.40 (1.25-9.22)*	*1.42 (0.68-2.97)*
Time spent at preschool between the ages of 3 and 6 years[e]				
Up to 15 hours/week	*1.00*	*1.00*	*1.00*	*1.00*
15-34 hours/week	*0.71 (0.34-1.48)*	*0.80 (0.41-1.54)*	*0.70 (0.27-1.84)*	*0.85 (0.45-1.62)*
≥35 hours/week	*1.00 (0.51-1.96)*	*0.56 (0.29-1.08)*	*0.91 (0.39-2.14)*	*0.68 (0.35-1.31)*
Does not remember	*2.83 (1.12-7.19)*	*1.89 (0.84-4.24)*	*8.00 (1.84-34.79)*	*2.50 (1.10-5.68)*

SDQ = Strengths and Difficulties Questionnaire; CES-DC = Center for Epidemiological Studies Depression Scale for Children; OR = odds ratio; CI = confidence intervals.

[a]*Propensity scores were estimated using the following covariates: maternal age at the child's birth, parental country of origin, education, income and employment in the child's first year of life and the child's gestational age, birth weight, being singleton and having a congenital malformation.*
[b]*Separate pairwise 1:1 propensity score matching analyses were performed for each exposure category with "≥20 months" as the reference group; the matched sample sizes were n =952 for 12-15 months and n =1022 for 16-19 months.*
[c]*Separate pairwise 1:1 propensity score-matched analyses were performed for each exposure category with "Up to 15 hours/week" as the reference group; the matched sample sizes were n =404 for "15–34 hours/week months", n =366 for "≥35 hours/week" and n=122 for "Does not remember".*
[d]*Separate pairwise 1:1 propensity score-matched analyses were performed for each exposure category with "Up to 15 hours/week" as the reference group; the matched sample sizes were n =436 for "15–34 hours/week months", n =434 for "≥35 hours/week" and n=108 for "Does not remember".*
[e]*Separate pairwise 1:1 propensity score-matched analyses were performed for each exposure category with "Up to 15 hours/week" as the reference group; the matched sample sizes were n =186 for "15–34 hours/week months", n =186 for "≥35 hours/week" and n=100 for "Does not remember".*

Please see Table S1 (Supplementary material)

Table S1 *Adjusted predicted probabilities and 95% confidence intervals of poor mental health outcomes by preschool-related variables*

Type of exposure	***Adjusted predicted probability, % (95% CI)***[a]			
	SDQ total score	***SDQ internalizing problems***	***SDQ externalizing problems***	***Depression measured by CES-DC***
The age when the child started preschool (n=2261)				
12-15 months	*19 (8-37)*	*24 (13-39)*	*16 (8-28)*	*22 (12-38)*
16-19 months	*15 (7-32)*	*23 (13-37)*	*11 (5-20)*	*21 (11-36)*
≥20 months	*14 (6-29)*	*22 (12-37)*	*11 (5-21)*	*22 (12-38)*
Time spent at preschool in different ages (hours/week)				
Time spent at preschool between the ages of 1 and 2 years (n=1779)[b]				
Up to 15 hours/week	*12 (4-29)*	*25 (12-45)*	*11 (4-24)*	*23 (11-42)*
15-34 hours/week	*13 (5-30)*	*22 (11-40)*	*9 (4-20)*	*22 (11-40)*
≥35 hours/week	*13 (5-30)*	*23 (11-41)*	*7 (3-17)*	*21 (10-38)*

Does not remember	*23 (8-49)*	*30 (14-53)*	*21 (8-43)*	*31 (14-55)*
Time spent at preschool between the ages of 2 and 3 years (n=2056)[c]				
Up to 15 hours/week	*11 (4-26)*	*20 (10-35)*	*8 (3-18)*	*18 (9-34)*
15-34 hours/week	*12 (5-27)*	*21 (11-35)*	*8 (3-17)*	*19 (10-34)*
≥35 hours/week	*15 (6-32)*	*23 (13-39)*	*9 (4-18)*	*21 (11-36)*
Does not remember	*31 (13-57)*	*32 (16-53)*	*23 (10-45)*	*33 (17-56)*
Time spent at preschool between the ages of 3 and 6 years (n=2208)[d]				
Up to 15 hours/week	*13 (5-31)*	*25 (12-43)*	*8 (3-18)*	*22 (11-41)*
15-34 hours/week	*12 (5-26)*	*22 (12-37)*	*9 (5-19)*	*20 (10-34)*
≥35 hours/week	*17 (7-34)*	*24 (13-39)*	*12 (6-23)*	*23 (13-39)*
Does not remember	*27 (11-53)*	*32 (16-54)*	*25 (11-46)*	*39 (20-62)*

SDQ = Strengths and Difficulties Questionnaire; CES-DC = Center for Epidemiological Studies Depression Scale for Children; CI = confidence intervals.

[a]*Adjusted for maternal age at child's birth, parental country of origin, education, income and employment in the child's first year of life and the child's gestational age, birth weight, being singleton and having a congenital malformation; some covariate categories were combined due to small cell counts.*

[b]*Analyses included study participants who started preschool between the ages 1-2 years.*

[c]*Analyses included study participants who started preschool before the age of 3 years.*

[d]*Analyses included study participants who had been at preschool.*

Please see the Statistical analysis section (page 9)

We assessed the model's assumptions for all main logistic regression analyses. We evaluated multicollinearity among covariates using generalized variance inflation factors; we did not find evidence of high multicollinearity. We conducted also influence diagnostic tests; we did not find influential observations.

Please see the Abstract section (page 3)

Conclusion: *Our findings suggest that starting preschool at the age of 12-15 months may be associated with poor mental health in adolescence, particularly with externalizing problems. Nonetheless, the results should be interpreted in light of the methodological constraints of observational studies, the multitude of our comparisons and the sample selection.*

Comment 5:

#Specific comments (page , raw)

p.1 Title: Do you mean "The effect of early preschool care on mental health in adolescence in Sweden. Results from the KUPOL longitudinal cohort"?

Response:

We have changed our title to "Preschool care early in life and mental health in adolescence in Sweden: a cohort study".

Comment 6:

p.3 r.15 Design should also include data sources

Response:

We specified in the Abstract section that we used data from the KUPOL study.

Please see the Abstract section (page 2)

Design: *A cohort study based on data from KUPOL (Swedish acronym for "Knowledge about Adolescents Mental Health and Learning").*

Comment 7:

p.3 r.17 Merge "Settings" with "Participants" and add more info

Response:

We merged the Setting and Participants sections.

Please see the Abstract section (page 2)

Setting and participants: *We used data from KUPOL, a longitudinal study conducted during 2013-2018, involving Swedish adolescents born 2000-2001. We included in the current analyses adolescents with available information on the exposures and the outcomes of interest (n=2261).*

Comment 8:

p.3 r.36 The statement emphasises on risk but then an Odds Ratio is reported. Although, this is not wrong it encourages wrong interpretation of Odds Ratios like Risk Ratios and that is

wrong. The same applies to the whole manuscript. You need to consider either reporting Risk Ratios instead or alter the interpretation to focus on odds rather than plain risk.

Response:

We have now carefully revised the manuscript to ensure that the terminology we used is consistent with the used statistical methods. We avoid the word "risk" when we refer to odds ratios.

Comment 9:

p.3 r.49 The conclusion statement reads very strong like an unambiguous public health call. Given the limited evidence from the reported p-values only in this analysis, the strong tone here is not justified. Try rephrasing or provide more robust evidence.

Response:

We have revised the conclusion to temper its message.

Please see the Abstract section (page 3)

Conclusion: *Our findings suggest that starting preschool at the age of 12-15 months may be associated with poor mental health in adolescence, particularly with externalizing problems. Nonetheless, the results should be interpreted in light of the methodological constraints of observational studies, the multitude of our comparisons and the sample selection.*

Comment 10:

p.3 r.57 Consider replacing "prevalence" with "frequency"

Response:

We deleted the corresponding sentence in the Conclusion.

Comment 11:

p.3 r.57-8 On what basis poor mental health in Sweden is justified. The statement reads problematic.

Response:

We deleted the corresponding sentence in the Conclusion.

Comment 12:

p.4 r.10 Remove "amount"

Response:

We have revised the sentence as suggested.

Please see the Strengths and Limitations of This Study section (page 3)

- *We investigated the association between the age at start of preschool and the time spent at preschool in different ages with several measures of mental health.*

Comment 13:

p.4 r.10 What means "with several dimensions"? Rephrase or remove

I would expect equal number of strength and limitation points

Response:

We have revised the sentence and added an additional limitation.

Please see the Strengths and limitations of this study (page 3)

- *We investigated the association between the age at start of preschool and the time spent at preschool in different ages with several measures of mental health.*
- *Our study included a contemporary cohort of Swedish children, allowing inference to present circumstances.*
- *We collected the information on exposure retrospectively, which may lead to some missclassification.*
- *Although we considered several confounders, we can not exclude the possibility of residual confounding by unmeasured factors.*

Comment 14:

p.5 r.27 The term "Western" is outdated, do you mean "high income" countries or developed?

Response:

We have revised the sentence as suggested.

Please see the Introduction section (page 4)

A further substantial societal change in the last decades is the "childcare transition",[4] *i.e. the increase in the proportion of women on the labor market during the late 70s and early 1980s in many high-income countries has led to an increase in the number children in community care at earlier and earlier ages.*[4 5]

Comment 15:

p.6 r.19 Do you mean "generously" subsidized?

Response:

We have revised the sentence as suggested.

Please see the Introduction section (page 5)

The generously subsidized daycare fees and several nationwide regulations make the quality of daycares within the country more homogeneous than in many other parts of the world; this may also result in the predictors of daycare utilization patterns, and hence the confounding in studies investigating its health effects, being different in Scandinavia than in countries with other sociopolitical contexts.[16 17]

Comment 16:

p.6 r.24 Do you mean "unique" around the world

Response:

We did not mean to imply that the Swedish daycare system is “unique” around the world. We wanted to highlight that the high level of public subsidy and nationwide regulation makes the daycare system in Sweden more uniform and differently structured compared with daycare systems in many other countries.

Comment 17:

p.7 r.31-32 The statement reads weak and vague. You need to briefly but clearly state the research hypotheses

Response:

We now present the study hypotheses.

Please see the Introduction section (page 6)

We hypothesized that an early start and a high number of weekly hours spent at preschool may increase the risk of poor mental health in adolescence.

Comment 18:

p.7 r.48 If the total population of the 541 schools is available, it should be added for transparency

"Study Population" The section repeats the word "study" in an unclear and misleading way. Do you mean that "longitudinal cohort study" is a "longitudinal electronic database" with various waves? The 3935 guardians gave consent to join your study or the KUPOL database? Please clarify and divide the section to "Data Source" and "(your) Study Population"

Response:

Unfortunately, we do not have information on the total eligible population in 541 schools. We have now divided the subsection into "Data source" and "Study population." We describe KUPOL as a longitudinal school-based cohort with repeated data collections, and clarify that the 3935 guardians provided consent for participation in the KUPOL cohort. We provide the flow chart for participation in the study in the Supplementary material.

Please see the Data source and study population section (pages 6-7)

Data source and study population

Data source. *The study was based on KUPOL (Swedish acronym for "Knowledge about Adolescents Mental Health and Learning"), a longitudinal cohort study involving adolescents born 2000-2001 from eight regions in Sweden.[30] Briefly, 541 secondary schools from central and Southern Sweden each having at least 20 students in each grade of the upper part of the primary school (i.e. grades 7-9) were asked to take part in the KUPOL study. The schools that accepted to participate (n=101, response rate at school level: 19%) provided students in the 7th grade in the academic years 2013/2014 and 2014/2015 (n=12512) and their families written information about the KUPOL study. Legal guardians of 3959 children (response rate at child level: 32%) gave written informed consent for participation in KUPOL. Data for wave 1 were collected when children were in grade 7, for wave 2 in grade 8, for wave 3 in grade 9 and for wave 4 in grade 10. Adolescents and their parents completed on each occasion extensive questionnaires concerning sociodemographic, school-related, lifestyle and psychosocial factors. We obtained further information from population-based registers for 3517 children, i.e., for whom we had written consent for register data retrieval.*

Study population. *Analyses for the current study were performed among adolescents whose parent responded to at least one question related to the child's preschool attendance and who*

had information on mental health from at least one wave in KUPOL (n=2261, i.e., 64% of the 3517). A flowchart of the participation in the study is included in the Supplementary material (Figure S1).

The study was approved by the Regional Ethical Review Board in Stockholm (reference numbers: 2012/2:11, 2016/1280-32).

Please see Figure S1 (Supplementary material)

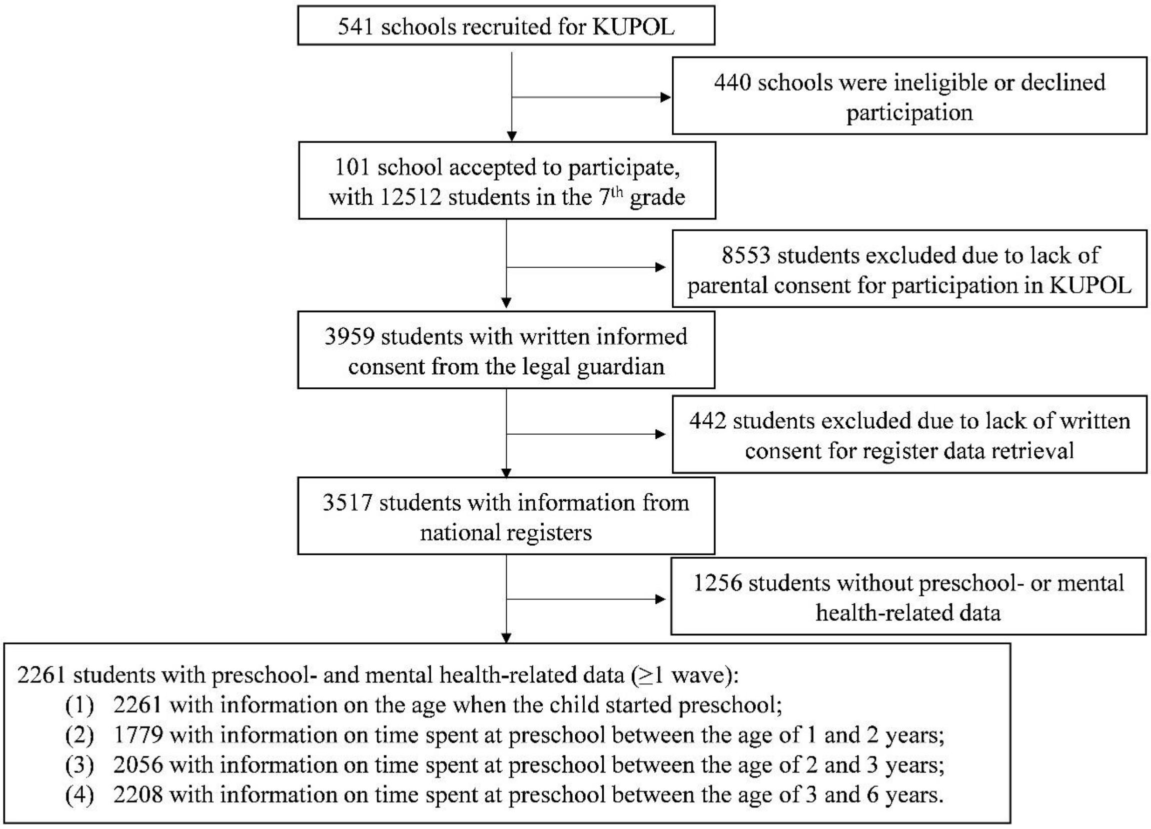

Figure S1 Flow chart for participation in the study. KUPOL = Swedish acronym for "Knowledge about Adolescents Mental Health and Learning".

Comment 19:

p.8 r.22 Replace "Measures" to read "Outcomes and Risk factors". Define and describe the study outcomes and the corresponding research questions. Explain the corresponding variables. Explain why all three are needed. Explain who collected SDQ and CES scales and how (postal, electronic?)

Response:

We have now replaced the *Measures* section by the *Study variables* section and re-structured this section. We now clearly define each study outcome and describe the variables used. We have also clarified who completed the SDQ and CES-DC and that both were collected within the KUPOL data collection. We have now revised the study questions and removed the question regarding daycare attendance as we did not have a univocal hypothesis for this research question.

Please see the Study variables section (pages 7-9)

Study variables

Exposure. *Information on preschool attendance was collected through the questionnaire sent to the legal guardians in wave 4. We studied two main exposures, i.e. (1) the age when the child started preschool or daycare (in months), and (2) the average weekly hours the child was at preschool or daycare between the ages 1-2, 2-3 and 3-6 years, with the answer possibilities for each age range being: up to 15 hours, 15-34 hours, ≥35 hours and "I don't remember". For the main analyses, we categorized the age when the child started preschool or daycare (in months) based on the tertile distribution as 12-15, 16-19 and ≥20 months.*

Outcomes. *Adolescents' mental health was measured on four occasions using the self-reported version of the Strengths and Difficulties Questionnaire (SDQ) and the Center for*

Epidemiological Studies Depression Scale for Children (CES-DC). The SDQ consists of 25 items that are grouped in five scales that assess emotional symptoms, hyperactivity/inattention symptoms, conduct problems, peer problems and prosocial behavior; responses are given on a three-point scale, i.e. not true (0), somewhat true (1) and certainly true (2).[31] *We calculated the total problem score by summing the responses on the 25 items, the internalizing problem score by summing scores on the emotional symptoms and the peer problems scales, and the externalizing problem score by summing answers on the hyperactivity/inattention and the conduct problems scales.*[32] *We categorized the scales based on the cutoffs suggested by Goodman et al.,*[32] *i.e. <18 versus ≥18 in case of the total difficulties score; <9 versus ≥9 in case of the internalizing problems scale and <11 versus ≥11 in case of the externalizing problems scale. The CES-DC includes 20 items and is used to screen for symptoms of depression among children and adolescents. Study participants rate on a scale ranging from 0 to 3 the frequency with which they experienced certain depressive symptoms in the past week. We calculated the total score by summing the responses on the individual items and categorized the variable as <30 versus ≥30, as previously suggested.*[33] *We categorized each outcome as scoring above the recommended cutoff at least on one of the four occasions versus never.*

Covariates*. Information on parents' education and country of birth, maternal age, child's gender, paternal and maternal income and employment status, gestational age, birth weight, singleton/multiple birth, child's congenital malformations, the quality of the parent-child relationship, confidence in others, peer network, family support, academic achievement were retrieved from questionnaires or registers, as described in the Supplementary material.*

Please see the Introduction section (page 6)

The aim of this study was to analyze whether the age at start of preschool and the average weekly hours at preschool are associated with mental ill-health among adolescents in Sweden.

Comment 20:

"Statistical Analysis"

The section needs careful re-writing with support from a qualified statistician as it reads vague at least. Analyses should match the study hypotheses, reported earlier and should be very specific and transparently presented

Response:

We have re-written the Statistical analysis section to improve clarity.

Please see the Statistical analysis section (pages 9-10)

Statistical analysis

We compared exposure groups by means of chi-square tests in case of categorical variables and by means of Kruskal-Wallis tests in case of continuous variables with non-normal distribution. We investigated the associations between the main preschool-related variables (i.e., age at start of preschool and time spent at preschool/week) and each mental health outcome (i.e., score above the cutoff on the total SDQ scale, SDQ internalizing problems, SDQ externalizing problems and CES-DC, respectively) using logistic regression. We adjusted for maternal age at child's birth, parental country of origin, education, income and employment in the child's first year of life, the child's gestational age, birth weight, being singleton and having a congenital malformation. The choice of confounders was based on (1) their known or theoretically plausible association with both the preschool-related variables and mental health and (2) not being on the causal pathway between exposure and outcome. We assessed the model's assumptions for all main logistic regression analyses. We evaluated multicollinearity among covariates using generalized variance inflation factors; we did not find evidence of high multicollinearity. We conducted also influence diagnostic tests; we did not find influential

observations. We estimated odds ratios (OR) and corresponding 95% confidence intervals (CI) and adjusted predictive probabilities. In case of missing covariates, in the main analyses we applied listwise deletion.

In case we observed an association, we further adjusted for factors that may be on the pathway between exposure and outcome, i.e. the warmth of the parent-child relationship, confidence in others, number of friends, family support and academic grades (one-by-one, all measured in wave 1) to investigate whether these factors may contribute to the explanation of the association of interest. To investigate whether the association of age at enrollment and time spent at preschool with mental health varies by the gender of the child (boy versus girl), maternal age when the child was born (categorized ≤30 versus ≥31 years) and maternal education (0-14 versus ≥15 years), we performed stratified analysis and formal tests of interaction with these variables.

We performed several sensitivity analyses. First, to balance baseline characteristics between exposure groups, we run analyses with propensity score matching (PSM). Second, to analyze whether our categorization of age at start of preschool influenced the results we repeated our main models with the variable treated as (1) continuous and (2) dichotomized at the median, i.e., at 18 months. Third, we repeated our main analyses with the outcome treated as continuous variable; we used linear regression to generate and compare least square means. Fourth, we imputed missing covariates using multiple imputation (20 imputations) and pooled the results using Rubin's rules.

All tests were two-sided, with α = 0.05.

SAS version 9.4 (SAS Institute, Inc) and R version 4.4 were used for the analyses.

Comment 21:

p.8 r.52 The introductory sentence reads weak and vague.

Response:

We have rewritten this sentence for clarity.

Please see the Statistical analysis section (page 9)

We investigated the associations between the main preschool-related variables (i.e., age at start of preschool and time spent at preschool/week) and each mental health outcome (i.e., score above the cutoff on the total SDQ scale, SDQ internalizing problems, SDQ externalizing problems and CES-DC, respectively) using logistic regression.

Comment 22:

p.8 r.54 The sentence should be removed and explained in the previous section (Outcomes and Risk Factors)

Response: We clarify the aim of our study in the Introduction.

Please see the Introduction section (page 6)

The aim of this study was to analyze whether the age at start of preschool and the average weekly hours at preschool are associated with mental ill-health among adolescents in Sweden.

Comment 23:

p.9 r.3 I do not advocate unadjusted analyses, especially within the public health perspective. Do not report unadjusted results in the main paper and tables.

Response:

We have now removed the results of unadjusted model from Table 2 and 3.

Comment 24:

p.9 r.10 Listwise deletion almost always increases bias. You need to check and discuss this. Why multiple imputation was not undertaken?

Response: We have now added analysis with multiple imputation as sensitivity analyses.

Please see the Statistical analysis section (page 10)

Fourth, we imputed missing covariates using multiple imputation (20 imputations) and pooled the results using Rubin's rules.

Please see Table S7 (Supplementary material)

Table S7 *Odds ratios and 95% confidence intervals for the association between preschool-related variables and mental health after multiple imputation for the covariates adjusted in the main model*[a]

Type of exposure	***Adjusted OR (95% CI)***[b]			
	SDQ total score	***SDQ internalizing problems***	***SDQ externalizing problems***	***Depression measured by the CES-DC***
The age when the child started preschool (n=2261)				
12-15 months	*1.36 (1.04-1.77)*	*1.05 (0.83-1.33)*	*1.45 (1.08-1.96)*	*1.04 (0.82-1.33)*
16-19 months	*1.07 (0.83-1.39)*	*1.03 (0.82-1.28)*	*0.97 (0.72-1.31)*	*0.90 (0.72-1.14)*
≥20 months	*1.00*	*1.00*	*1.00*	*1.00*
Time spent at preschool in different ages (hours/week)				
Time spent at preschool between the ages of 1 and 2 years (n=1779)[c]				
Up to 15 hours/week	*1.00*	*1.00*	*1.00*	*1.00*
15-34 hours/week	*1.07 (0.76-1.52)*	*0.89 (0.66-1.19)*	*0.87 (0.60-1.26)*	*0.94 (0.69-1.29)*
≥35 hours/week	*1.16 (0.76-1.76)*	*0.88 (0.61-1.26)*	*0.88 (0.55-1.40)*	*0.87 (0.60-1.28)*
Does not remember	*1.77 (1.02-3.07)*	*1.35 (0.82-2.23)*	*1.89 (1.07-3.35)*	*1.51 (0.91-2.52)*
Time spent at preschool between the ages of 2 and 3 years (n=2056)[d]				
Up to 15 hours/week	*1.00*	*1.00*	*1.00*	*1.00*
15-34 hours/week	*1.10 (0.78-1.56)*	*1.21 (0.89-1.63)*	*0.84 (0.58-1.22)*	*1.13 (0.82-1.55)*
≥35 hours/week	*1.41 (0.96-2.08)*	*1.29 (0.92-1.82)*	*1.14 (0.76-1.73)*	*1.25 (0.88-1.77)*
Does not remember	*2.70 (1.53-4.74)*	*1.93 (1.13-3.29)*	*2.35 (1.30-4.22)*	*1.76 (1.01-3.05)*
Time spent at preschool between the ages of 3 and 6 years (n=2208)[e]				
Up to 15 hours/week	*1.00*	*1.00*	*1.00*	*1.00*
15-34 hours/week	*1.02 (0.62-1.68)*	*0.82 (0.55-1.24)*	*1.29 (0.71-2.33)*	*0.96 (0.62-1.48)*
≥35 hours/week	*1.40 (0.85-2.31)*	*0.90 (0.59-1.36)*	*1.73 (0.95-3.16)*	*1.07 (0.68-1.68)*
Does not remember	*2.24 (1.14-4.41)*	*1.37 (0.75-2.52)*	*3.34 (1.57-7.13)*	*1.79 (0.95-3.34)*

SDQ = Strengths and Difficulties Questionnaire; CES-DC = Center for Epidemiological Studies Depression Scale for Children; OR = odds ratio; CI = confidence intervals.

[a]*Multiple imputation was made for maternal age at child's birth, parental country of origin, education, income and employment in the child's first year of life and the child's gestational age, birth weight, being singleton and having a congenital malformation.*

[b]*Adjusted for maternal age at child's birth, parental country of origin, education, income and employment in the child's first year of life and the child's gestational age, birth weight, being singleton and having a congenital malformation.*
[c]*Analyses included study participants who started preschool between the ages 1-2 years.*
[d]*Analyses included study participants who started preschool before the age of 3 years.*
[e]*Analyses included study participants who had been at preschool.*

Comment 25:

p.9 r.12-31 The paragraph needs adequate discussion of the model(s) used and all adjusting variables in each model and the reasoning behind them. You should better consider report Risk Ratios (a GLM with log-link function instead of the logit-link function) as well as marginal probabilities that can quantify the risk appropriately and meaningfully.

Response:

We have extended the Statistical analysis section to provide more information about our models and the rationale behind our covariate selection. We used logistic regression as the primary analytic approach given the binary outcome and its widespread use in epidemiological studies. We now report also adjusted marginal probabilities.

Please see the Statistical analysis section (page 9)

We investigated the associations between the main preschool-related variables (i.e., age at start of preschool and time spent at preschool/week) and each mental health outcome (i.e., score above the cutoff on the total SDQ scale, SDQ internalizing problems, SDQ externalizing problems and CES-DC, respectively) using logistic regression.

Please see the Statistical analysis section (page 9)

The choice of confounders was based on (1) their known or theoretically plausible association with both the preschool-related variables and mental health and (2) not being on the causal pathway between exposure and outcome.

Please see Table S1 (Supplementary material)

Table S1 *Adjusted predicted probabilities and 95% confidence intervals of poor mental health outcomes by preschool-related variables*

Type of exposure	***Adjusted predicted probability, % (95% CI)[a]***			
	SDQ total score	***SDQ internalizing problems***	***SDQ externalizing problems***	***Depression measured by CES-DC***
The age when the child started preschool (n=2261)				
12-15 months	*19 (8-37)*	*24 (13-39)*	*16 (8-28)*	*22 (12-38)*
16-19 months	*15 (7-32)*	*23 (13-37)*	*11 (5-20)*	*21 (11-36)*
≥20 months	*14 (6-29)*	*22 (12-37)*	*11 (5-21)*	*22 (12-38)*
Time spent at preschool in different ages (hours/week)				
Time spent at preschool between the ages of 1 and 2 years (n=1779)[b]				
Up to 15 hours/week	*12 (4-29)*	*25 (12-45)*	*11 (4-24)*	*23 (11-42)*
15-34 hours/week	*13 (5-30)*	*22 (11-40)*	*9 (4-20)*	*22 (11-40)*
≥35 hours/week	*13 (5-30)*	*23 (11-41)*	*7 (3-17)*	*21 (10-38)*
Does not remember	*23 (8-49)*	*30 (14-53)*	*21 (8-43)*	*31 (14-55)*
Time spent at preschool between the ages of 2 and 3 years (n=2056)[c]				
Up to 15 hours/week	*11 (4-26)*	*20 (10-35)*	*8 (3-18)*	*18 (9-34)*
15-34 hours/week	*12 (5-27)*	*21 (11-35)*	*8 (3-17)*	*19 (10-34)*
≥35 hours/week	*15 (6-32)*	*23 (13-39)*	*9 (4-18)*	*21 (11-36)*
Does not remember	*31 (13-57)*	*32 (16-53)*	*23 (10-45)*	*33 (17-56)*
Time spent at preschool between the ages of 3 and 6 years (n=2208)[d]				
Up to 15 hours/week	*13 (5-31)*	*25 (12-43)*	*8 (3-18)*	*22 (11-41)*
15-34 hours/week	*12 (5-26)*	*22 (12-37)*	*9 (5-19)*	*20 (10-34)*
≥35 hours/week	*17 (7-34)*	*24 (13-39)*	*12 (6-23)*	*23 (13-39)*
Does not remember	*27 (11-53)*	*32 (16-54)*	*25 (11-46)*	*39 (20-62)*

SDQ = Strengths and Difficulties Questionnaire; CES-DC = Center for Epidemiological Studies Depression Scale for Children; CI = confidence intervals.

[a]Adjusted for maternal age at child's birth, parental country of origin, education, income and employment in the child's first year of life and the child's gestational age, birth weight, being singleton and having a congenital malformation; some covariate categories were combined due to small cell counts.
[b]Analyses included study participants who started preschool between the ages 1-2 years.
[c]Analyses included study participants who started preschool before the age of 3 years.
[d]Analyses included study participants who had been at preschool.

Comment 26:

p.9 r.38 A CONSORT-type diagram about the detailed identification of the study sample should be provided with justification about the mismatch between 3959 consented guardians and 2261 analysable(?).

Response:

We now added a flow chart of participation in the study in the Supplementary material.

Please see Figure S1 (Supplementary material)

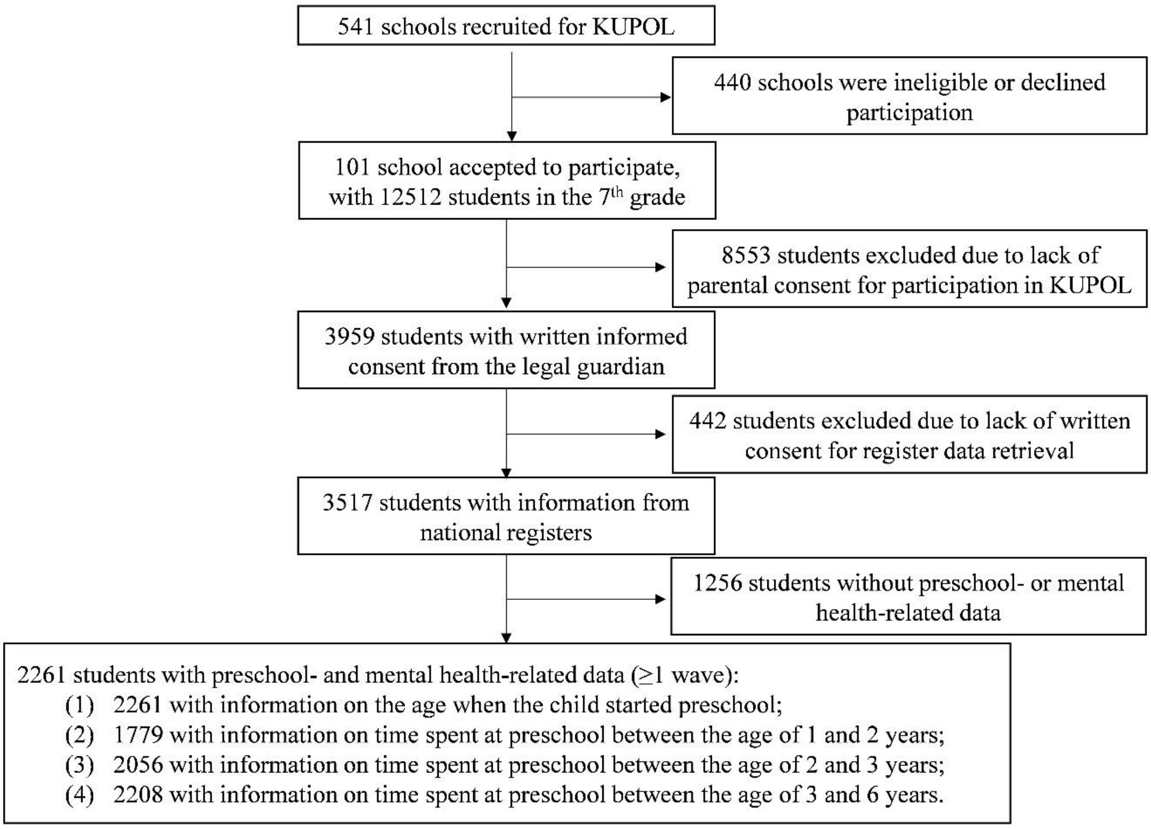

Figure S1 Flow chart for participation in the study. KUPOL = Swedish acronym for "Knowledge about Adolescents Mental Health and Learning".

Comment 27:

p9. r.47 Table1 misses p-values as this is not a randomised study.

Response:

We have now added in Table 1 p-values comparing study variables according to exposure to age at start of preschool.

Comment 28:

p.9-10 The findings need to be verified with sensitivity analyses and the results should be properly reported in tables that mirror the current table 1. In other words, tables should follow the 3 group mode as in the current table 1.

Response:

We would like to clarify that the age at start of preschool was consistently categorized into three groups throughout the analyses. Table 1 focuses on a single exposure and therefore presents exposure groups in rows. In contrast, Tables 2 and 3 include multiple exposures simultaneously and different populations, i.e. those at risk, which required to present exposures in columns rather than rows. We now add several additional analyses, as suggested.

Please see the Statistical analysis section (page 10)

We performed several sensitivity analyses. First, to balance baseline characteristics between exposure groups, we run analyses with propensity score matching (PSM). Second, to analyze whether our categorization of age at start of preschool influenced the results we repeated our main models with the variable treated as (1) continuous and (2) dichotomized at the median,

i.e., at 18 months. Third, we repeated our main analyses with the outcome treated as continuous variable; we used linear regression to generate and compare least square means. Fourth, we imputed missing covariates using multiple imputation (20 imputations) and pooled the results using Rubin's rules.

Comment 29:

p.10 r.54-

The discussion (initial) subsection reads unreasonably strong given the inherent bias of the study. The remaining part is fine.

Th list of limitations should also include no use of proper group-balancing methods such as PSM and/or IPW for causal inference through eliminating selection bias.

Response:

We have revised the discussion to have a more balanced interpretation of the findings. As mentioned above, we have now added also the results of the PSM analyses.

Please see the Discussion section (page 12)

Swedish children who started preschool at the age of 12-15 months had higher odds of high total SDQ and externalizing problems scores than those who started preschool at 20 months or later. We found no association between the age at start of preschool and the odds of internalizing problems, nor between the number of hours spent at preschool in different ages and the odds of mental ill-health.

Comment 30:

p.14 r.3 Unclear what the call for prospective studies means here, as in public health topics like this, prospective and efficient designs of this scale are almost never feasible. For this reason, the Real World Evidence methodology has been developed for the last 20 years in order to allow causal inference.

Response:

We have removed the corresponding sentence and revised the Discussion section to avoid overly strong interpretations and to better reflect the limitations of our study.

Please see the Discussion section (page 17)

Nonetheless, these results need to be interpreted in light of the methodological constraints of observational studies, the multitude of comparisons and the sample selection.

Comment 31:

p.14 Ethics approval dated and numbered should be add somewhere.

Response: We have now added information on the authority that gave ethical approval for the study and the approval numbers.

Please see the Data source and study population section (page 7)

The study was approved by the Regional Ethical Review Board in Stockholm (reference numbers: 2012/2:11, 2016/1280-32).

Comment 32:

main Tables: They read unconventional and cluttered.

Response:

To improve clarity, we have removed the results for preschool attendance and of the unadjusted models and improved the format (in Tables 2 and 3).

Comment 33:

T1: column1 should be titled as "characteristics". Remove "exposure". Should have four columns as group1,2,3 and then total and then p-values

The 3-group frequencies should exact match the total. This is not the case here.

Response:

We have revised Table 1 as suggested.

Comment 34:

T2: The table should be restructured to follow the previous table format. Then, risk estimated and 95% CIs should be carefully reported for the adjusted models only, and in a transparent way. Missing values should also be reported clearly. All models should be fully adjusted of all available covariates. All covariates should be stated at the "Methods" section once. Overall SDQ scores per group should also be provided in the table.

Response:

Table 2 and 3 differs in structure from Table 1 because Tables 2 and 3 present associations for multiple exposures and outcomes simultaneously, which precludes arranging exposure groups as rows in the same way as in Table 1.

All models were adjusted for the same set of covariates, which are now listed once in the Methods section. We have now clarified in the footnotes of Tables 2 and 3 that analyses were restricted to participants with complete data on the exposure, outcome and the adjusted covariates.

We have performed sensitivity analysis to calculate least square means from linear regression analyses with the continuous mental health scores.

Please see the Statistical analysis section (page 9)

We adjusted for maternal age at child's birth, parental country of origin, education, income and employment in the child's first year of life, the child's gestational age, birth weight, being singleton and having a congenital malformation.

Please see Table 2 (pages 26-27)

Table 2 *Odds ratios and 95% confidence intervals for the association between preschool-related variables and mental health as measured by the Strengths and Difficulties Questionnaire*

Type of exposure	***Total n (%)***	***SDQ total score***		***SDQ internalizing problems***		***SDQ externalizing problems***	
		Events	***Adjusted OR (95% CI)[a]***	***Events***	***Adjusted OR (95% CI)[a]***	***Events***	***Adjusted OR (95% CI)[a]***
The age when the child started preschool (n=2261)							
12-15 months	*642 (28.4)*	*150*	*1.39 (1.02-1.90)*	*196*	*1.09 (0.83-1.43)*	*120*	*1.52 (1.08-2.16)*
16-19 months	*869 (38.4)*	*163*	*1.10 (0.82-1.49)*	*255*	*1.02 (0.79-1.32)*	*112*	*0.99 (0.70-1.39)*
≥20 months	*750 (33.2)*	*134*	*1.00*	*215*	*1.00*	*100*	*1.00*
Time spent at preschool in different ages (hours/week)							
Time spent at preschool between the ages of 1 and 2 years (n=1779)[b]							
Up to 15 hours/week	*271 (15.2)*	*51*	*1.00*	*84*	*1.00*	*46*	*1.00*
15-34 hours/week	*1090 (61.3)*	*208*	*1.09 (0.73-1.63)*	*312*	*0.85 (0.61-1.20)*	*147*	*0.84 (0.55-1.28)*
≥35 hours/week	*323 (18.2)*	*67*	*1.09 (0.68-1.77)*	*94*	*0.87 (0.58-1.31)*	*45*	*0.65 (0.38-1.13)*
Does not remember	*95 (5.3)*	*27*	*2.14 (1.11-4.10)*	*35*	*1.28 (0.70-2.34)*	*26*	*2.16 (1.11-4.20)*
Time spent at preschool between the ages of 2 and 3 years (n=2056)[c]							
Up to 15 hours/week	*275 (13.4)*	*49*	*1.00*	*71*	*1.00*	*44*	*1.00*
15-34 hours/week	*1231 (59.9)*	*227*	*1.11 (0.75-1.64)*	*359*	*1.05 (0.75-1.47)*	*160*	*0.93 (0.61-1.43)*
≥35 hours/week	*471 (22.9)*	*107*	*1.39 (0.90-2.15)*	*146*	*1.20 (0.83-1.76)*	*77*	*1.04 (0.64-1.70)*
Does not remember	*79 (3.8)*	*29*	*3.52 (1.83-6.78)*	*31*	*1.84 (0.98-3.46)*	*25*	*3.38 (1.71-6.67)*
Time spent at preschool between the ages of 3 and 6 years (n=2208)[d]							
Up to 15 hours/week	*119 (5.4)*	*22*	*1.00*	*40*	*1.00*	*14*	*1.00*
15-34 hours/week	*1180 (53.4)*	*208*	*0.88 (0.51-1.51)*	*333*	*0.86 (0.54-1.38)*	*155*	*1.29 (0.66-2.52)*
≥35 hours/week	*833 (37.7)*	*182*	*1.32 (0.76-2.29)*	*247*	*0.97 (0.60-1.57)*	*129*	*1.76 (0.89-3.47)*
Does not remember	*76 (3.4)*	*25*	*2.46 (1.14-5.34)*	*30*	*1.45 (0.71-2.99)*	*23*	*4.09 (1.71-9.76)*

SDQ = Strengths and Difficulties Questionnaire; OR = odds ratio; CI = confidence intervals.

[a]Adjusted for maternal age at child's birth, parental country of origin, education, income and employment in the child's first year of life and the child's gestational age, birth weight, being singleton and having a congenital malformation; analyses were restricted to participants with complete data on the exposure, outcome and adjusted covariates.
[b]Analyses included study participants who started preschool between the ages 1-2 years.
[c]Analyses included study participants who started preschool before the age of 3 years.
[d]Analyses included study participants who had been at preschool.

Please see Table 3 (page 28)

Table 3 *Odds ratios and 95% confidence intervals for the association between preschool-related variables and depression as measured by the Center for Epidemiological Studies Depression Scale for Children*

Type of exposure[b]	Total n (%)	Depression measured by CES-DC	
		Events	Adjusted OR (95% CI)[a]
**The age when the child started preschool (n=2261)**			
12-15 months	642 (28.4)	175	1.00 (0.76-1.33)
16-19 months	869 (38.4)	210	0.91 (0.70-1.19)
≥20 months	750 (33.2)	198	1.00
**Time spent at preschool in different ages (hours/week)**			
Time spent at preschool between the ages of 1 and 2 years (n=1779)[b]			
Up to 15 hours/week	271 (15.2)	71	1.00
15-34 hours/week	1090 (61.3)	269	0.96 (0.67-1.37)
≥35 hours/week	323 (18.2)	76	0.87 (0.56-1.34)
Does not remember	95 (5.3)	33	1.52 (0.82-2.80)
Time spent at preschool between the ages of 2 and 3 years (n=2056)[c]			
Up to 15 hours/week	275 (13.4)	63	1.00
15-34 hours/week	1231 (59.9)	304	1.06 (0.74-1.50)
≥35 hours/week	471 (22.9)	126	1.17 (0.78-1.74)
Does not remember	79 (3.8)	27	2.25 (1.20-4.24)
Time spent at preschool between the ages of 3 and 6 years (n=2208)[d]			
Up to 15 hours/week	119 (5.4)	31	1.00
15-34 hours/week	1180 (53.4)	288	0.84 (0.51-1.37)
≥35 hours/week	833 (37.7)	218	1.05 (0.64-1.74)
Does not remember	76 (3.4)	29	2.23 (1.08-4.60)

CES-DC = Center for Epidemiological Studies Depression Scale for Children; OR = odds ratio; CI = confidence intervals.

[a]Adjusted for maternal age at child's birth, parental country of origin, education, income and employment in the child's first year of life and the child's gestational age, birth weight, being singleton and having a congenital malformation; analyses were restricted to participants with complete data on the exposure, outcome and adjusted covariates.

[b]Analyses included study participants who started preschool between the ages 1-2 years.

[c]Analyses included study participants who started preschool before the age of 3 years.

[d]Analyses included study participants who had been at preschool.

Please see Table S6 (Supplementary material)

Table S6 *Least square means and 95% confidence intervals for scores on the Strengths and Difficulties Questionnaire and on the Center for Epidemiological Studies Depression Scale for Children according to preschool-related variables*

Type of exposure	***SDQ total score***		***SDQ internalizing problems***		***SDQ externalizing problems***		***Depression measured by CES-DC***	
	LS Mean (95% CI)[a]	***P-value***	***LS Mean (95% CI)[a]***	***P-value***	***LS Mean (95% CI)[a]***	***P-value***	***LS Mean (95% CI)[a]***	***P-value***
The age when the child started preschool (n=2261)								
12-15 months	*13 (11-15)*	*0.03*	*7 (6-8)*	*0.53*	*7 (6-8)*	*0.02*	*21 (17-24)*	*0.50*
16-19 months	*12 (11-14)*	*0.77*	*7 (6-8)*	*0.99*	*6 (5-7)*	*0.61*	*20 (17-24)*	*0.84*
≥20 months	*12 (11-14)*	*-*	*7 (6-8)*	*-*	*6 (5-7)*	*-*	*20 (17-24)*	*-*
Time spent at preschool in different ages (hours/week)								
Time spent at preschool between the ages of 1 and 2 years (n=1779)[b]								
Up to 15 hours/week	*13 (11-15)*	*-*	*7 (5-8)*	*-*	*7 (6-8)*	*-*	*20 (16-24)*	*-*
15-34 hours/week	*12 (10-14)*	*0.33*	*6 (5-8)*	*0.37*	*7 (5-8)*	*0.36*	*20 (15-24)*	*0.68*
≥35 hours/week	*13 (11-15)*	*0.68*	*7 (5-8)*	*0.87*	*7 (5-8)*	*0.42*	*20 (16-24)*	*0.90*
Does not remember	*15 (12-17)*	*0.01*	*8 (6-9)*	*0.05*	*8 (7-9)*	*0.03*	*23 (18-28)*	*0.05*
Time spent at preschool between the ages of 2 and 3 years (n=2056)[c]								
Up to 15 hours/week	*12 (10-14)*	*-*	*6 (5-7)*	*-*	*6 (5-7)*	*-*	*19 (16-23)*	*-*
15-34 hours/week	*12 (11-14)*	*0.45*	*6 (5-7)*	*0.67*	*6 (5-7)*	*0.77*	*20 (17-23)*	*0.40*
≥35 hours/week	*13 (11-14)*	*0.06*	*7 (6-8)*	*0.22*	*7 (6-8)*	*0.18*	*20 (17-24)*	*0.22*
Does not remember	*15 (13-17)*	*0.00*	*8 (6-9)*	*0.01*	*8 (7-9)*	*0.00*	*25 (21-30)*	*0.00*
Time spent at preschool between the ages of 3 and 6 years (n=2208)[d]								
Up to 15 hours/week	*12 (10-14)*	*-*	*6 (5-7)*	*-*	*6 (5-8)*	*-*	*20 (16-24)*	*-*
15-34 hours/week	*12 (10-13)*	*0.56*	*6 (5-7)*	*0.91*	*6 (5-7)*	*0.86*	*19 (15-22)*	*0.29*
≥35 hours/week	*13 (11-14)*	*0.27*	*7 (6-8)*	*0.29*	*7 (6-8)*	*0.17*	*21 (17-24)*	*0.83*
Does not remember	*15 (12-17)*	*0.01*	*7 (6-9)*	*0.03*	*8 (7-9)*	*0.01*	*25 (21-30)*	*0.01*

SDQ = Strengths and Difficulties Questionnaire; CES-DC = Center for Epidemiological Studies Depression Scale for Children; LS Mean=least square mean; CI=confidence interval.

[a]*Adjusted for maternal age at child's birth, parental country of origin, education, income and employment in the child's first year of life and the child's gestational age, birth weight, being singleton and having a congenital malformation; analyses were restricted to participants with complete data on the exposure, outcome and adjusted covariates.*
[b]*Analyses included study participants who started preschool between the ages 1-2 years.*
[c]*Analyses included study participants who started preschool before the age of 3 years.*
[d]*Analyses included study participants who had been at preschool.*

Comment 35:

T3: The table should follow the same structure as tables 1 and 2. Transparent reporting of the estimates should be provided.

Response:

Please see the response to comment 28 and comment 34.

Comment 36:

Supplement Tables

Overall, the tables follow the expected structure reporting estimates by group but almost all of them are of minor or no importance for a public health reader. One or two maximum could be available in a supplement. Why the group m=20 is the reference group?

Response:

According to your suggestion, we have now removed several tables from the Supplementary material. Age at start above 20 months was selected as the reference category to ensure comparability between the sensitivity and main analyses.

Comment 37:

S1: Report only full-model estimates. Is the "N" column for total frequencies? How that "total" applies to both groups in the table? Personally, I like the no-reporting of any p-values here.

Response:

N is the number of participants without missing data on each variable used and without missing data on exposure, outcome and adjusted covariates. We clarified this in the table footnote.

Please see Table S2 (Supplementary material)

Table S2. *Associations between age when the child started preschool and high total strengths and difficulties score, before and after adjustment for potential explanatory variables assessed at age 13*

Potential explanatory variables	*N[a]*	*The age when the child started preschool[b]*			
		12-15 months		***16-19 months***	
		OR (95 % CI) in the base model[c]	***OR (95 % CI) in the base model[c] + potential explanatory factor***	***OR (95 % CI) in the base model[c]***	***OR (95 % CI) in the base model[c] + potential explanatory factor***
Maternal warmth as rated by the child	*1716*	*1.37 (1.00-1.87)*	*1.32 (0.96-1.81)*	*1.10 (0.82-1.48)*	*1.09 (0.81-1.48)*
Paternal warmth as rated by the child	*1715*	*1.39 (1.02-1.90)*	*1.31 (0.95-1.81)*	*1.10 (0.81-1.48)*	*1.10 (0.81-1.49)*
Parental warmth as rated by the parent	*1719*	*1.39 (1.02-1.90)*	*1.39 (1.02-1.90)*	*1.10 (0.82-1.49)*	*1.10 (0.82-1.49)*
Confidence in others	*1718*	*1.39 (1.02-1.90)*	*1.37 (1.00-1.89)*	*1.10 (0.82-1.48)*	*1.14 (0.84-1.54)*
Number of male friends	*1691*	*1.35 (0.99-1.85)*	*1.37 (1.00-1.88)*	*1.08 (0.80-1.46)*	*1.10 (0.81-1.48)*
Number of female friends	*1686*	*1.38 (1.01-1.89)*	*1.38 (1.01-1.90)*	*1.10 (0.82-1.49)*	*1.11 (0.82-1.50)*
Family support in school-related issues	*1718*	*1.38 (1.01-1.88)*	*1.29 (0.94-1.79)*	*1.10 (0.82-1.49)*	*1.12 (0.82-1.53)*
Academic results	*1598*	*1.34 (0.96-1.86)*	*1.33 (0.95-1.85)*	*1.11 (0.81-1.51)*	*1.15 (0.84-1.58)*

OR = odds ratio; CI = confidence intervals.

[a]The number of participants without missing data on exposure, outcome and covariates.

[b]The age ≥20 months was considered as the reference.

[c]Adjusted for maternal age at child's birth, parental country of origin, education, income and employment in the child's first year of life and the child's gestational age, birth weight, being singleton and having a congenital malformation.

Comment 38:

S2 and S3: The table reports some large ORs with corresponding confidence intervals that does not include 1 (e.g. Girl) but there is a p-value next to them that comes from the interaction term. This is a quite misleading display. Please try a better display for those results, if of importance. Textual only reporting somewhere in the Results section should be preferable.

Response:

We have removed Tables S2 and S3 and now mention results regarding effect modification only in the text of the Results section.

Comment 39:

S4 and S5: Heavily cluttered tables with loads of estimates with p-values here and there. I cannot see where those tables are discussed in the manuscript.

Response:

We have deleted Tables S4 and S5.

Comment 40:

Supplement Information

The section is useful but too lengthy and should be truncated by 15% at least.

Response:

We have now removed Tables S2-S5 from the Supplementary material and shortened the text regarding the description of the study variables.

Reviewer #2:Dr. Björn Högberg, Umea Universitet

Comments to the Author:

The paper addresses a substantively important topic, which is commendable. The topic is also politically controversial, which should by no means discourage scholars from studying it, but it does mean that extra caution may be required in interpreting the results.

Response:

Thank you for the positive comment on our work.

Comment 1:

My main objection is that the paper, like many papers in public health and the social sciences, wavers between being causal and associational/descriptive in focus. The language is clearly associational, and causal terms are carefully avoided when describing the results. However, the research question is undoubtedly motivated from a causal perspective: an association between childcare and mental health is not very interesting in itself. Moreover, the introduction and discussion are largely devoted to topics that implicitly assume causal relationships (discussion of policy in the intro, mechanisms on p 11). Finally, the authors adjust for several (I think nine) confounders, and covariate/confounder adjustment is typically motivated by reducing bias (and hence to capture identify effects; however, the authors do not explicitly motivate why they include covariates in this study).

What I am getting at is that I think that the paper needs a more elaborate discussion of causality and confounding/bias. This is not merely something that should only be mentioned as a limitation; readers deserve to know to what extent the odds ratios can be given a causal interpretation, and what the main sources of bias may be. In particular, I think it would be highly useful with an elaborate discussion concerning selection into preschool and especially early preschool. Who are the parents who decide to put their child into preschool at an early age? What are their reasons for doing so? I can imagine that parents are more prone to send their child to preschool early if they have had a difficult first year, either because the child is "turbulent", cries a lot or is hyperactive or because the parents have had difficulties and are emotionally exhausted (e.g., maternal depression). I can also imagine that parents with financial difficulties are less likely to stay at home with the child for a long time because they cannot afford to do so. Finally, I can imagine that parents with progressive parenting values are more likely to send their child to preschool early compared to parents with more conservative values, and the progressive parents may also be more tolerant of externalizing behaviors and/or more open about discussing internalizing problems with the child. These are just some ideas: the point is that the authors should carefully think through what kind of social processes that they are trying to capture, and also guide the readers in this context. The authors can actually partly investigate this themselves using their data. What differences do they observe across exposure groups, and what do these differences tell them about possible sources of bias?

Response:

We have now extended the discussion with alternative explanations for the observed association. In particular, we now include a more detailed discussion of factors related to

selection into early entry in preschool, including parental socioeconomic circumstances, child characteristics and parenting values.

Please see the Discussion section (pages 14-15)

Several methodological aspects need to be addressed when interpreting the observed associations in addition to the possibility for a causal explanation. The first one is selection. Parents who choose to enroll their children in daycare at early ages and their children may differ from their counterparts enrolling in daycare in later ages on personal characteristics and contextual factors that make it difficult to make predictions on the magnitude and directions of the potential bias. For example, some parents may have chosen an early enrolment in daycare because of the child's behavioral problems (e.g., frequent crying or being hyperactive), and/or because of the parent himself/herself had poor mental health. On the other hand, socially well integrated parents with progressive parenting values may send their children to daycare earlier than parents who do not endorse trust in the community services; the former may also be more tolerant to externalizing behaviours in the child. Despite of the rather comprehensive attempt to control for confounders we cannot exclude the possibility that the modest association between start of daycare at the age of 12-15 months and the odds of high SDQ and high externalizing problems in adolescence may be due to bias.

Comment 2:

A second major concern: The stated aim is to investigate "whether age at starting preschool and weekly hours spent at preschool in different ages are associated with mental ill-health". This is an omnibus aim (or possible two aims: one for each exposure) that is tested using four different outcome variables and different types of exposure variables. In total, tables 2 and 3

include 32 odds ratios that may be said to investigate this aim (I here count the adjusted odds ratios for the different categories of age of starting and time at preschool for the four outcomes, and exclude the unadjusted odds ratios and the "does not remember" category). 2 of these 32 odds ratios are statistically significant, and in both cases the CI is quite close to overlapping with 1. Strictly speaking, the authors should probably apply multiple testing correction given the large number of tests of the same research question (or implicit null hypothesis). A standard Bonferroni correction would then render both significant effects non-significant. Now, multiple testing correction is partly a philosophical issue, and something I do not always practice myself. The main point is that the results can be interpreted in two radically different ways: (1) as showing that early preschool starting age is associated with (some) mental health problems, based on the two significant odds ratios, or (2) as showing that the evidence for an association between preschool attendance (starting age and hour/week) and mental health is fairly weak, fragile, and model dependent; the second interpretation also accounts for the 30 or so non-significant odds ratios. My reading of the results is that the second interpretation is more reasonable and better aligned with the combined evidence presented in tables 2 and 3. I also think that the second interpretation is supported by the rather weak effects of the two significant odds ratios (both having CIs almost overlapping with 1, meaning that a small amount of unobserved confounding would render them nonsignificant) and by the inconsistencies of the results: If early preschool attendance truly causes mental health problems, then we should expect to observe a dose-response relationship between number of hours in age 1-2 and mental health problems, but we don't in this case (the effects of hours are non-significant, and the point estimates are actually in the reverse direction for three out of four outcomes). The meaning of these inconsistencies should also be discussed in the paper.

Response:

We have now revised our discussion regarding the possible explanations of the association, including those you suggested.

Please see the Discussion section (page 15)

Second, if the association between early start of daycare and the risk of high SDQ and high externalizing problems was causal we could expect a dose-response association between an increasing number of hours spent at daycare, especially between the ages 1-2 years and the risks of these outcomes, which was not supported by the data. Third, given the multitude of tests, some of the observed statistically significant associations could be due to chance.

Comment 3:

*A partly related comment concerns the sample. The authors touch upon the non-response rate and representativeness of the sample in the limitations-section. However, I think this is potentially a bigger issue that should be introduced already on p 6 ("Study population"). 541 schools were sampled, 101 of which participated. That's a school-level response-rate of less than 20%. Of the 12 512 students in these schools, 3 959 provided consent (or rather: their guardians did), which is 32 %. Of the 3 959 who consented, 2 563 provided data on mental health, and somewhat fewer also on the pre-school variables. Then we are down at an individual-level response rate of around 20%. Assuming that the number of students does not vary between responding and non-responding schools (I have no idea if this is the case, but just for the sake of the argument), this gives a total response rate of 4% (0.2*0.2=0.04).*

This final sample is probably selected, and possibly heavily so, in numerous ways. Such a response rate requires a careful discussion of representativeness, selection, bias and so on. Admittedly, such a discussion is partly provided in the limitations-section, but I feel that it

perhaps deserves even greater attention (not merely as a limitation, but also concerning the implications).

Additionally, the authors have access to register data that can be linked to the survey. Is it possible to construct weights based on register data, such that groups with low response-rates are given a greater weight?

Response:

We now describe the multi-stage response rate more explicitly in the Data source and Study population section, and add a flow chart of participants in the Supplementary material. We have revised the conclusions and note that the results need to be interpreted in light of the fact that the sample was selected.

We could unfortunately not use register data to weight the sample to construct non-response weights; register data are available only for individuals included in the KUPOL dataset and for whom we had informed consent for register linkage.

Please see the Data source and study population section (pages 6-7)

Data source and study population

Data source. *The study was based on KUPOL (Swedish acronym for "Knowledge about Adolescents Mental Health and Learning"), a longitudinal cohort study involving adolescents born 2000-2001 from eight regions in Sweden.*[30] *Briefly, 541 secondary schools from central and Southern Sweden each having at least 20 students in each grade of the upper part of the primary school (i.e. grades 7-9) were asked to take part in the KUPOL study. The schools that accepted to participate (n=101, response rate at school level: 19%) provided students in the 7th grade in the academic years 2013/2014 and 2014/2015 (n=12512) and their families written information about the KUPOL study. Legal guardians of 3959 children (response rate at child*

level: 32%) gave written informed consent for participation in KUPOL. Data for wave 1 were collected when children were in grade 7, for wave 2 in grade 8, for wave 3 in grade 9 and for wave 4 in grade 10. Adolescents and their parents completed on each occasion extensive questionnaires concerning sociodemographic, school-related, lifestyle and psychosocial factors. We obtained further information from population-based registers for 3517 children, i.e., for whom we had written consent for register data retrieval.

Study population. *Analyses for the current study were performed among adolescents whose parent responded to at least one question related to the child's preschool attendance and who had information on mental health from at least one wave in KUPOL (n=2261, i.e., 64% of the 3517). A flowchart of the participation in the study is included in the Supplementary material (Figure S1).*

Please see Figure S1(Supplementary material)

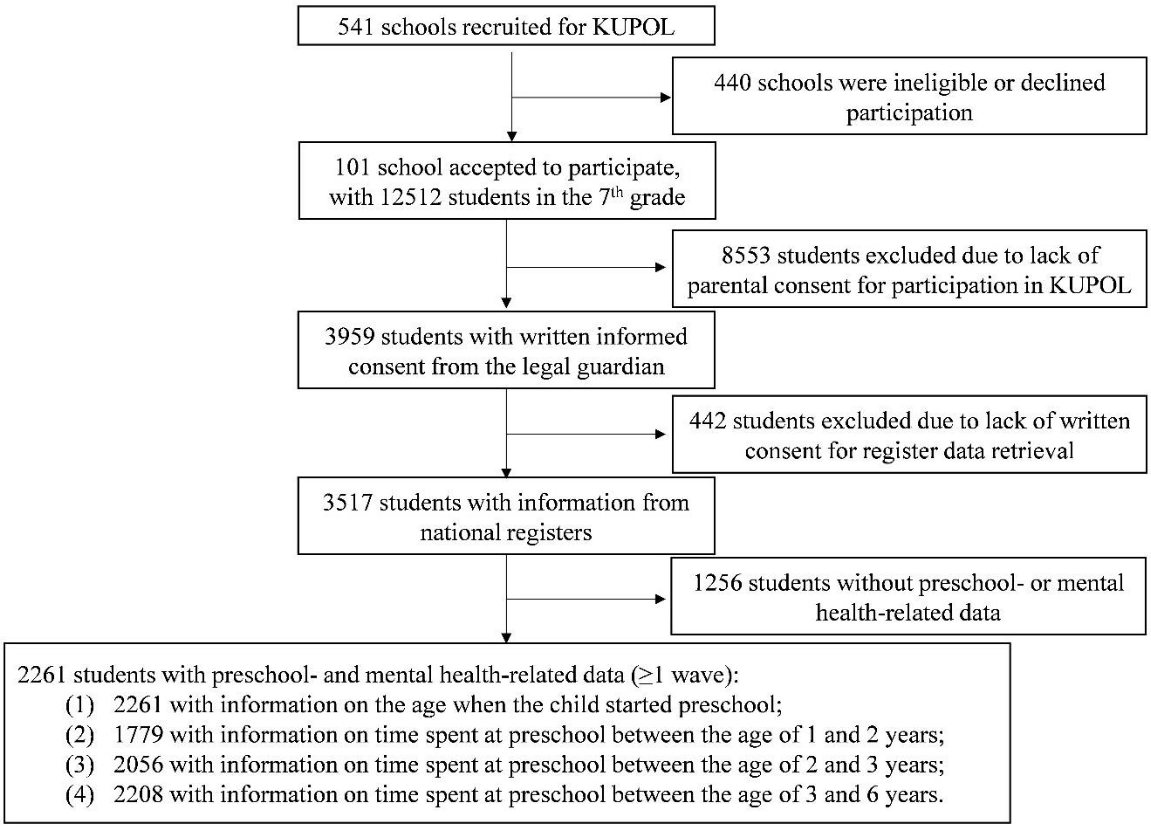

Figure S1 Flow chart for participation in the study. KUPOL = Swedish acronym for "Knowledge about Adolescents Mental Health and Learning".

Please see the conclusion (page 17)

Nonetheless, these results need to be interpreted in light of the methodological constraints of observational studies, the multitude of comparisons and the sample selection.

Comment 4:

Minor comments

P 12: "Several earlier studies have suggested that there may be some benefit for enrolling early in a high quality daycare for children from disadvantaged as opposed to those from more advantaged families. It is therefore plausible that associations observed in our study may be underestimated, given the protective factors associated with a highly educated and Swedish-born population, i.e. categories that are overrepresented in our sample."

I don't follow how the second sentence builds on the first. If the type of children that gain from attending preschool early (the disadvantaged) are underrepresented, then the associations are more likely to be overestimated, not underestimated, right? Or am I missing something here?

Response:

We have revised the text as described below.

Please see the Discussion section (page 16)

Several earlier studies have suggested that there may be some benefit for enrolling early in a high-quality daycare for children from disadvantaged as opposed to those from more advantaged families,[7] suggesting that the association between early enrollment in daycare and

mental health varies by parental socioeconomic status. Given the characteristics of the participants in this study, it is plausible that the adverse or null associations observed are overestimated compared to what we would have observed in the total population where a substantial proportion of children would have been affected positively by early enrolment in day care. Nevertheless, stratified analyses by parental education and country of birth did not suggest evidence of effect modification by these factors.

Comment 5:

P 7: "A detailed description of the outcomes and the covariates is provided in the Supplementary material." I agree that detailed descriptions can be included in the supplement. However, readers of the main manuscript would gain from having some notion of what covariates are included, and why. Two or three sentences would suffice.

Response:

We have now modified the structure of the Study variables section, as suggested.

Please see the Study variables section (page 9)

***Covariates**. Information on parents' education and country of birth, maternal age, child's gender, paternal and maternal income and employment status, gestational age, birth weight, singleton/multiple birth, child's congenital malformations, the quality of the parent-child relationship, confidence in others, peer network, family support, academic achievement were retrieved from questionnaires or registers, as described in the Supplementary material.*

Comment 6:

The paper is mostly well-written and easy to follow. There are some errors that could be picked up by a careful proof-reading, however. Some examples: P 9, line 12: "There was no an association between…". Either it is "no" association, or "an" association? P 10, line 42: "A study by Hagekull and Bohlin". "By" shouldn't be in superscript.

Response:

We have carefully proofread the manuscript and corrected the identified typos.

Comment 7:

P 2 of the supplement: "Information on paternal and maternal income and employment status during the year after childbirth …" Income and employment the year after childbirth may not be very meaningful considering that many parents are on parental leave then, and thus have lower incomes and possibly no employment. Moreover, these measures may increase rather than reduce bias since they are differentially associated with age of preschool attendance. Parents who send their children to preschool early will likely have higher incomes, all else being equal, because they can go back to work.

Response:

We chose this measure of income before exposure as it would be a better determinant of time of starting preschool than the income before birth, which for women is affected by health during pregnancy and the associated sick leave and other social insurance benefits. We expected that in all families one of the parents would be on parental leave before the child's first birthday,

i.e. the familial income loss due to parental leave would affect virtually all children before the age of 1.

Comment 8:

I may come about as too harsh here: I do commend the authors for studying a substantively important issue with important policy implications, which unfortunately is not always the case in public health research. I also think that the manuscript is well-written and the analyses are sound. I would like to see the manuscript published in some form. However, I'm not sure if I agree with the author's main take-away of the paper, and I think causality should be addressed head-on (given the policy implications of the findings).

Response:

We have now revised the discussion to give a more balanced interpretation based on the points raised.

VERSION 2 - REVIEW

**Reviewer**	**1**
**Name**	**bouliotis, george**
**Affiliation**	**University of Warwick Warwick Clinical Trials Unit, Warwick Medical School**
**Date**	**03-Feb-2026**
**COI**	

I want to thank the team for the effort to come back with an improved version of the manuscript. I acknowledge the improvement and the clarity in some previously problematic parts as well as the additional analyses and results provided this time, using decluttered tables.

My critique below is around the conclusion from this research the authors state twice in the updated manuscript in page13 lines 15-20 and in page 17 lines 12-17. The authors interpret their results as early pre-schooling is a high public health risk for mental health, whereas the displayed results do not indicate any such risk. In particular:

1. From the reported numbers in the table 2, it is quickly estimated that the Risk Difference (RD) between the base-category (20m) and the early-category (12m) is only 5.5% (unadjusted OR=1.40) regarding the SDQ total. Similarly, the RD for externalising problems is 5.4% (unadjusted OR=1.49). Such a small difference in the risk of (higher) odds is practically null in a pragmatic context, especially after taking into account the serious limitations of this research.

2. The CES-DC scale indicates, within its lower confidence interval, a somewhat 30% benefit for the 16m category and a 26% benefit for the 12m category

3. The PSM table, if correct, returned ORs that are below 1.50 whereas ORs above 2 or better 3 would be reasonable for public health policy changes and actions. It is noticeable that only the "externalised problems" result remained statistically significant, indicating very slight increase in odds (not in relative risk!) that is of no real concern, but rather as small statistical effect.

3. The Least Square Means table that are displayed for first time, as authors did not present unadjusted means, indicate that the average SDQ total score in the cohort is only 13 for the early group, far below their chosen cutoff value of 18. Given the substantial overlap of all three confidence intervals, it is boldly suggested that no real difference of concern really exists.

4. The Predicted Probabilities, if correctly estimated, once again suggest that for the early group the probability of having higher than 18 SDQ score is only 19% whereas the probability for late and very late groups are about 15%, and when checking the groups' upper CIs their difference is again 5%

5. The grouping of the age (key) variable to questionable timing as month 15 and month 19 is behind the inflated ratios. It is noticeable that when a continuous, instead of categorical, age in months variable was used in the model, the ratio is about 1 indicating that schooling age has clearly no effect on odds

6. The sample diagram as well as the missingness rate and more importantly the hidden missingness behind the labelling "cannot remember" strongly indicates that the results are not generalisable. As a result, no public health message is justified by the results.

**Reviewer**	**2**
**Name**	**Högberg, Björn**
**Affiliation**	**Umea Universitet, Social Work**
**Date**	**23-Jan-2026**
**COI**	

I commend the authors on their thorough review of the paper. Most of my comments have been satisfactorily addressed. I want to push a little bit more on my previous major comments, though:

I think that the response concerning multiple tests is somewhat insufficient: (p 16) “Third, given the multitude of tests, some of the observed statistically significant associations could be due to chance”. This reads as an acknowledgment of a potential limitation; however, I think that the issue is much more profound than that (which was also the point with my previous comment, although it perhaps did not come across clearly enough). I think it is the responsibility of the authors to provide a balanced assessment of where the weight of the evidence point. The authors “hypothesized that an early start and a high number of weekly hours spent at preschool may increase the risk of poor mental health in adolescence”. Given what they found (assuming that the hypothesis was formulated before looking at the results), do they think that the hypothesis is verified or rejected? Or do they think that the evidence is more rather than less supportive of the hypothesis? My reading of tables 2 and 3 is that the weight of the evidence suggests that neither an early start nor a high number of weekly hours is (causally) associated with mental health. I reach this assessment based on (1) the overwhelming number of null-findings compared to significant effects (suggesting that the two significant effects are most likely flukes / statistical noise); and (2) that a logical and parsimonious implication of a causal effect is a dose-response relationship, which is not observed here. This is of course a matter of interpretation and judgement, not objective truth, and my interpretation may not be the best or the only one. However, I do think that reception of the study is crucially dependent on how the authors weigh the evidence, and which evidence they emphasise. Right now the emphasis is overwhelmingly on the small minority of significant estimates, not on the large majority of non-significant ones (where, moreover, in many cases the point estimates are also in the “wrong” direction from the perspective of the hypothesis). This is not a balanced assessment or framing in my view.

In light of this, I also think that the conclusion in the abstract is too strong, or perhaps rather focuses on the wrong side of the coin. A more balanced conclusion would be that the study found weak and inconsistent evidence of an association, but that the association between age and externalizing problems may deserve further investigation and monitoring.

For editors: please note that I find the "Review Checklist" rather unhelpful. Thus, editors should not place much weight on my replies there.

VERSION 2 - AUTHOR RESPONSE

Reviewer #1: Dr. George Bouliotis, University of Warwick Warwick Clinical Trials Unit

Comment 1:

Comments to the Author:

I want to thank the team for the effort to come back with an improved version of the manuscript. I acknowledge the improvement and the clarity in some previously problematic parts as well as the additional analyses and results provided this time, using decluttered tables.

My critique below is around the conclusion from this research the authors state twice in the updated manuscript in page13 lines 15-20 and in page 17 lines 12-17. The authors interpret their results as early pre-schooling is a high public health risk for mental health, whereas the displayed results do not indicate any such risk. In particular:

1. From the reported numbers in the table 2, it is quickly estimated that the Risk Difference (RD) between the base-category (20m) and the early-category (12m) is only 5.5% (unadjusted OR=1.40) regarding the SDQ total. Similarly, the RD for externalising problems is 5.4% (unadjusted OR=1.49). Such a small difference in the risk of (higher) odds is practically null in a pragmatic context, especially after taking into account the serious limitations of this research.

2. The CES-DC scale indicates, within its lower confidence interval, a somewhat 30% benefit for the 16m category and a 26% benefit for the 12m category

3. The PSM table, if correct, returned ORs that are below 1.50 whereas ORs above 2 or better 3 would be reasonable for public health policy changes and actions. It is noticeable that only the "externalised problems" result remained statistically significant, indicating very slight increase in odds (not in relative risk!) that is of no real concern, but rather as small statistical effect.

3. The Least Square Means table that are displayed for first time, as authors did not present unadjusted means, indicate that the average SDQ total score in the cohort is only 13 for the

early group, far below their chosen cutoff value of 18. Given the substantial overlap of all three confidence intervals, it is boldly suggested that no real difference of concern really exists.

4. The Predicted Probabilities, if correctly estimated, once again suggest that for the early group the probability of having higher than 18 SDQ score is only 19% whereas the probability for late and very late groups are about 15%, and when checking the groups' upper CIs their difference is again 5%

5. The grouping of the age (key) variable to questionable timing as month 15 and month 19 is behind the inflated ratios. It is noticeable that when a continuous, instead of categorical, age in months variable was used in the model, the ratio is about 1 indicating that schooling age has clearly no effect on odds

6. The sample diagram as well as the missingness rate and more importantly the hidden missingness behind the labelling "cannot remember" strongly indicates that the results are not generalisable. As a result, no public health message is justified by the results.

Response:

We have revised the Discussion section, including the conclusions, to give a more balanced interpretation of the findings of the study and its conclusion, considering that associations were null to modest, the absolute risk differences and population attributable risks were low and that we had multiple comparisons. For revisions, please see our Word track changes in the Abstract, Methods, Results and in the first and last paragraphs of the Discussions section.

Reviewer #2: Dr. Björn Högberg, Umea Universitet

Comment:

Comments to the Author:

I commend the authors on their thorough review of the paper. Most of my comments have been satisfactorily addressed. I want to push a little bit more on my previous major comments, though:

I think that the response concerning multiple tests is somewhat insufficient: (p 16) "Third, given the multitude of tests, some of the observed statistically significant associations could be due to chance". This reads as an acknowledgment of a potential limitation; however, I think that the issue is much more profound than that (which was also the point with my previous comment, although it perhaps did not come across clearly enough). I think it is the responsibility of the authors to provide a balanced assessment of where the weight of the evidence point. The authors "hypothesized that an early start and a high number of weekly hours spent at preschool may increase the risk of poor mental health in adolescence". Given what they found (assuming that the hypothesis was formulated before looking at the results), do they think that the hypothesis is verified or rejected? Or do they think that the evidence is more rather than less supportive of the hypothesis? My reading of tables 2 and 3 is that the weight of the evidence suggests that neither an early start nor a high number of weekly hours is (causally) associated with mental health. I reach this assessment based on (1) the overwhelming number of null-findings compared to significant effects (suggesting that the two significant effects are most likely flukes / statistical noise); and (2) that a logical and parsimonious implication of a causal effect is a dose-response relationship, which is not observed here. This is of course a matter of interpretation and judgement, not objective truth, and my interpretation may not be the best or the only one. However, I do think that reception

of the study is crucially dependent on how the authors weigh the evidence, and which evidence they emphasise. Right now the emphasis is overwhelmingly on the small minority of significant estimates, not on the large majority of non-significant ones (where, moreover, in many cases the point estimates are also in the "wrong" direction from the perspective of the hypothesis). This is not a balanced assessment or framing in my view.

In light of this, I also think that the conclusion in the abstract is too strong, or perhaps rather focuses on the wrong side of the coin. A more balanced conclusion would be that the study found weak and inconsistent evidence of an association, but that the association between age and externalizing problems may deserve further investigation and monitoring.

For editors: please note that I find the "Review Checklist" rather unhelpful. Thus, editors should not place much weight on my replies there.

Response:

Though we are aware of and concerned of the problem of multiple comparisons we would not fully guide our overall interpretation of the findings based on the *proportion* of statistically significant associations among all performed tests. There are several reasons to this. First, we had clear a priori hypotheses, formulated by the first author in 2010 − i.e. years before the KUPOL study's data collection started − based on theories in this area, prior literature and biological plausibility. These hypotheses, along with the knowledge gap outlined in the manuscript, led to proposing to study this research question to the KUPOL team and a decision to include the preschool-related questions in the KUPOL parental questionnaire.

Second, we think it is important to consider that the high number of analyses are "clustered" and would not contribute as separate, independent entities to the denominator when estimating the "proportion of positive associations", of which around 5% may be expected to be due to chance. In our view the results within the different clusters and the comparisons of results across the clusters may be used to scrutinize if they confirm and reinforce each other or not. For example, the *outcomes* are not independent of each other. The SDQ internalizing problems scale and the CES-DC capture similar phenomena; the CES-DC questionnaire was included in the KUPOL questionnaire in addition to the SDQ internalizing scale as it is considered to assess depressive symptoms better than the SDQ internalizing scale. The consistency of results across these two scales is reassuring. Furthermore, the SDQ total score is calculated by combining information from the SDQ internalizing and externalizing subscales. We interpret that the association observed between early start of preschool and the externalizing problems also drives and probably explains the association for the total SDQ score. Similarly, the three large set of analyses according to the *time spent at preschool* (in ages 1-2, 2-3 and 3-6 years) are also strongly interrelated; if there is no association with high number of hours spent at preschool in ages 1-2 years it may be reasonable to expect that there will be no association later on, a presumption in line with our findings. Further *misclassification of exposure* is likely to be higher and *statistical power* lower in case of the variable regarding number of hours spent at preschool in different ages than in case of the age at enrolling in preschool. Further, parents have a higher short-term flexibility to adopt weekly time of use of preschool services according to the child's socioemotional reactions than what they may have with the age of start of preschool. These may contribute to the explanation of the differences in findings according to main exposure groups. Similarly, several of the *sensitivity analyses* were conducted with the aim to test the robustness of specific findings from the main analyses; results of several of these sensitivity analyses align with their correspondent from the main analyses. If chance was the

only potential explanation for the results, we might expect a more random pattern of findings and less in line with what was described above. As in any observational study, we are not able to establish causality.

Nevertheless, we agree with the need of a more balanced interpretation of the results. In line with the suggestion of reviewer 1, we now place more emphasis on the potential public health impact of the findings. We report and discuss also absolute risk differences and population-attributable fractions for the associations between age at start of preschool and SDQ total score and SDQ externalizing score. These measures suggest a limited public health implication for our findings. We have revised the manuscript, including its conclusions, to provide a more balanced interpretation, considering both reviewers' and our interpretation of the results.

For revisions – all aimed to address this point – please see our Word track changes in the Abstract, Methods, Results and Discussion sections.
